# Hdac3 regulates lymphovenous and lymphatic valve formation

**DOI:** 10.1172/JCI92852

**Published:** 2017-10-16

**Authors:** Harish P. Janardhan, Zachary J. Milstone, Masahiro Shin, Nathan D. Lawson, John F. Keaney, Chinmay M. Trivedi

**Affiliations:** 1 Division of Cardiovascular Medicine,; 2 Department of Medicine, and; 3 Department of Molecular, Cell, and Cancer Biology, University of Massachusetts Medical School, Worcester, Massachusetts, USA.

**Keywords:** Development, Vascular Biology, Epigenetics, Lymph, endothelial cells

## Abstract

Lymphedema, the most common lymphatic anomaly, involves defective lymphatic valve development; yet the epigenetic modifiers underlying lymphatic valve morphogenesis remain elusive. Here, we showed that during mouse development, the histone-modifying enzyme histone deacetylase 3 (Hdac3) regulates the formation of both lymphovenous valves, which maintain the separation of the blood and lymphatic vascular systems, and the lymphatic valves. Endothelium-specific ablation of Hdac3 in mice led to blood-filled lymphatic vessels, edema, defective lymphovenous valve morphogenesis, improper lymphatic drainage, defective lymphatic valve maturation, and complete lethality. Hdac3-deficient lymphovenous valves and lymphatic vessels exhibited reduced expression of the transcription factor Gata2 and its target genes. In response to oscillatory shear stress, the transcription factors Tal1, Gata2, and Ets1/2 physically interacted with and recruited Hdac3 to the evolutionarily conserved E-box–GATA–ETS composite element of a Gata2 intragenic enhancer. In turn, Hdac3 recruited histone acetyltransferase Ep300 to form an enhanceosome complex that promoted Gata2 expression. Together, these results identify Hdac3 as a key epigenetic modifier that maintains blood-lymph separation and integrates both extrinsic forces and intrinsic cues to regulate lymphatic valve development.

## Introduction

Thin-walled lymphatic capillaries collect interstitial fluid (lymph) and transport it via collecting lymphatic vessels to the thoracic duct, which in turn drains into the subclavian vein (1). Improper drainage of this extravasated protein-rich fluid from the tissues causes it to accumulate, resulting in lymphedema (2). Intraluminal lymphatic valves within the collecting lymphatic vessels and bicuspid lymphovenous valves ensure anterograde lymph drainage into the venous circulation (3). Additionally, these valves prevent backflow of venous blood into the thoracic and right lymphatic ducts, effectively separating the blood and lymphatic systems (4, 5). Platelet-mediated intervascular hemostasis also functions with lymphovenous valves to maintain this separation throughout life (4). Developmental or functional defects in these valves can cause both primary and secondary lymphedema (6, 7).

Lymphatic endothelial cells (LECs), the building blocks of the mammalian lymphatic vasculature, experience shear stress generated by the cephalad movement of lymphatic fluid (8). Recent evidence demonstrates that this oscillatory lymph flow–mediated shear stress initiates stepwise morphological and molecular changes within LECs that lead to the formation of lymphatic valves (8–11). Specifically, oscillatory shear stress (OSS) induces the expression of genes, including *GATA2*, *FOXC2*, *GJA4*, and *ITGA9*, in LECs that are important for lymphatic valve development (9, 10, 12). GATA2, an upstream transcriptional regulator of *FOXC2*, *PROX1*, *GJA4*, and *ITGA9,* is important for blood-lymph separation and the development of lymphovenous and lymphatic valves (9, 10, 13, 14). Despite this evidence, the mechanisms driving OSS-mediated GATA2 expression during lymphatic valve development remain elusive.

GATA2 belongs to an evolutionarily conserved family of zinc finger transcription factors that play important roles in diverse developmental programs (15, 16). Monoallelic missense mutations and intragenic microdeletions in human *GATA2* cause Emberger syndrome, characterized by primary lymphedema with a predisposition to myelodysplastic syndrome or acute myeloid leukemia (17–19). Moreover, 2 recurrent mutations that cause reduced GATA2 expression (c.1017+512del28 and c.1017+572C>T) within a highly conserved 167-bp intragenic enhancer of intron 5 of *GATA2* have been found in patients with primary lymphedema (20–22). Recent studies in transgenic mice demonstrate that this intragenic enhancer confers GATA2 expression specifically within endothelial cells of the lymphatic, cardiac, and vascular systems (14, 23). Indeed, murine embryos lacking this Gata2 intragenic enhancer have reduced Gata2 expression and phenocopy the endothelial knockout of Gata2 (24). Thus, there is strong evidence that reduced GATA2 expression leads to lymphedema (10, 20, 25).

Broadly, enhancers function as *cis*-regulatory elements controlling gene expression in a spatiotemporal and cell type–specific manner (26). Enhancer element activation is dependent on recruitment of specific transcription factors, coactivators, chromatin remodelers, and histone-modifying enzymes (27). The latter establish histone marks that often serve to recruit a multitude of transcription factors and cofactors to gene regulatory elements (26, 28). In addition, active enhancers show enrichment of Lys-27 acetylation on histone H3 (H3K27ac) and occupancy of the histone acetyltransferase EP300 (29–31). While the complex regulation of enhancer elements is starting to be defined, the role of histone-modifying factors in regulating the transcriptional activity of the Gata2 intragenic enhancer remains unknown.

Two opposing classes of histone-modifying enzymes, histone acetyltransferases (HATs) and histone deacetylases (HDACs), regulate the acetylation state of histones within an enhancer (32). Acetylation by HATs is generally associated with transcriptional activation, while HDAC-mediated deacetylation usually results in transcriptional repression (32). On the basis of their catalytic mechanism and sequence homology, HDACs are classified into 5 subfamilies: class I (Hdac1, 2, 3, and 8), class IIa (Hdac4, 5, 7, and 9), class IIb (Hdac6 and 10), class III (Sirt1, 2, 3, 4, 5, 6, and 7), and class IV (Hdac11) (33). Among HDACs, global loss of class I HDACs in mice causes embryonic or neonatal lethality, suggesting that these enzymes play pivotal roles in development (34–38). Recent studies from our laboratory and others have identified unique and tissue-specific functions of ubiquitously expressed class I HDACs during cardiac and craniofacial development (36, 39–42). Class I HDACs lack intrinsic DNA-binding domains and are instead recruited to the chromatin in a signal-dependent manner via their interactions with multiple protein nuclear complexes, transcription factors, and cofactors (32). Contrary to previous assumptions, emerging data from genome-wide mapping reveal that the majority of HATs and class I HDACs are recruited to promoter and enhancer regions of actively transcribed genes with acetylated histones (43). Among class I HDACs, Hdac3 mainly occupies intragenic and intergenic regions, including enhancers marked by H3K27ac and EP300 enrichment (44–47). Despite advances in understanding the developmental roles of class I HDACs, their role in vascular and lymphatic development remains undefined.

Here, we show that endothelial Hdac3, but not Hdac1 or Hdac2, is important for blood lymphatic separation. Mice lacking endothelial Hdac3 demonstrate congenital lymphedema due to defective lymphovenous and intraluminal lymphatic valve development. Our studies reveal that Hdac3-mediated epigenetic regulation of an OSS-dependent Gata2 intragenic enhancer orchestrates lymphatic valve development and establishes blood-lymph separation.

## Results

### Lymphatic endothelial Hdac3 regulates blood-lymph separation.

**Figure 1:**
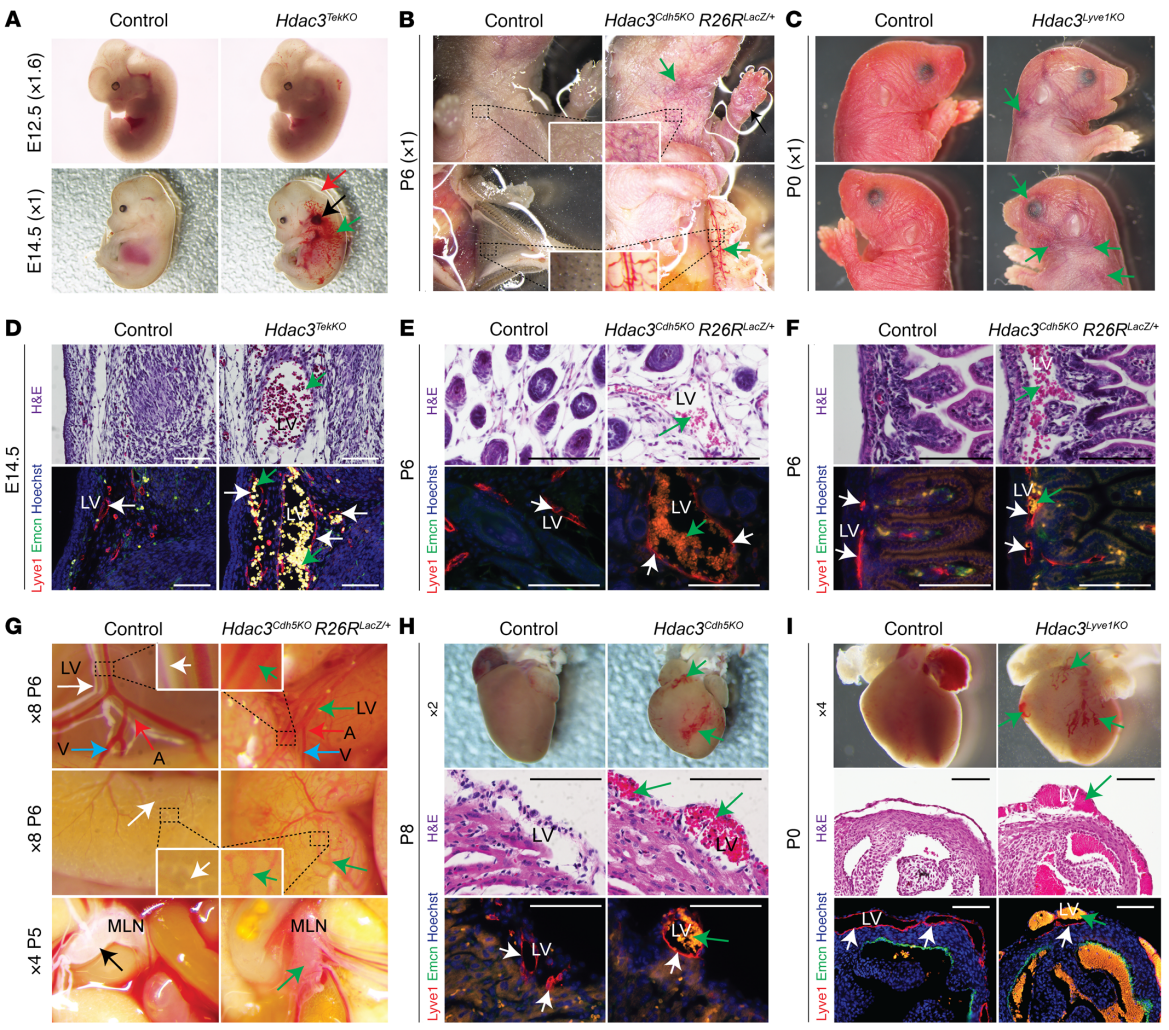
Lymphatic endothelial Hdac3 regulates blood-lymphatic separation. (**A**) Dissected E12.5 and E14.5 *Hdac3^TekKO^* embryos. Green arrow shows ectatic superficial vessels; black arrow shows pooling of blood in the jugular region; red arrow shows swelling. (**B** and **C**) Neonatal (P6) *Hdac3^Cdh5KO^* mice (**B**) and P0 *Hdac3^Lyve1KO^* mice (**C**) had abnormal blood-filled dermal vessels (green arrows) compared with controls. (**D**–**F**) H&E and coimmunofluorescence staining for Lyve1 (lymphatic marker, red) and Emcn (venous marker, green) shows blood-filled (green arrows) dermal lymphatic vessels in E14.5 *Hdac3^TekKO^* murine embryos (**D**) and blood-filled dermal (**E**) and intestinal lymphatic vessels (**F**) in P6 *Hdac3^Cdh5KO^* neonates (**E** and **F**). White arrows show lymphatic vessels. (**G**) Dissected intestine of control and *Hdac3^Cdh5KO^* P6 neonates. White, red, and blue arrows indicate lymphatic, arterial, and venous vessels, respectively; black arrow shows a mesenteric lymph node; green arrows show blood-filled lymphatic vessels and a mesenteric lymph node in *Hdac3^Cdh5KO^* P6 neonates. (**H** and **I**) P8 *Hdac3^Cdh5KO^* (**H**) and P0 *Hdac3^Lyve1KO^* (**I**) hearts show ectatic and hemorrhagic superficial vessels (green arrows). H&E and coimmunofluorescence staining for Lyve1 (lymphatic marker, red) and Emcn (venous marker, green) shows blood-filled (green arrows) cardiac lymphatic vessels in P8 *Hdac3^Cdh5KO^* (**H**) and P0 *Hdac3^Lyve1KO^* (**I**) murine hearts. White arrows show lymphatic vessels. Scale bars: 100 μm. A, artery; LV, lymphatic vessel; MLN, mesenteric lymph node; V, vein. See also Supplemental Figures 1–4 and Supplemental Tables 1–5.

Hdac3 is ubiquitously expressed, including within LECs of developing lymphovenous valves, mesenteric lymphatic vessels and valves, heart, and peripheral lymphatic vessels (Supplemental Figure 1, A–G; supplemental material available online with this article; https://doi.org/10.1172/JCI92852DS1) (39, 40, 48, 49). Germline deletion of *Hdac3* results in embryonic lethality before E9.5 (34, 37). To determine the function of Hdac3 in the developing blood and lymphatic vasculature, we deleted *Hdac3* in endothelial cells using *Hdac3^fl/fl^* mice and 3 Cre lines: *Tek*-*Cre* (*Hdac3^TekKO^*), *Cdh5*-*Cre* (*Hdac3^Cdh5KO^*), and *Lyve1-Cre* (*Hdac3^Lyve1KO^*) (Supplemental Figure 1, A, D, and G) (50–52). *Hdac3^TekKO^* mice were identified until E14.5 but not at birth (P0), indicating complete embryonic lethality (Supplemental Tables 1 and 2). *Hdac3^TekKO^* embryos showed ectatic superficial vessels, pooling of blood in the jugular region, and severe edema at E14.5 compared with that seen in E12.5 embryos (Figure 1A). *Hdac3^Cdh5KO^* and *Hdac3^Lyve1KO^* neonates revealed similar ectatic dermal vessels at P6 and P0 and neonatal lethality at P9 and P0, respectively (Figure 1, B and C, and Supplemental Tables 3 and 4). Endothelial cells lining blood-filled superficial vessels in *Hdac3^TekKO^* embryos and *Hdac3^Cdh5KO^* neonates were positive for the lymphatic marker Lyve1, but negative for the venous marker Emcn, suggesting lymphatic identity (Figure 1, D and E). Additionally, *Hdac3^Cdh5KO^* neonates exhibited blood-filled lymphatic vessels in intestine and mesentery between P5 and P6 and in heart at P0 (Figure 1, F–H). Similarly, *Hdac3^Lyve1KO^* neonates had blood-filled cardiac lymphatic vessels at P0 (Figure 1I). We observed no apparent structural defects in *Hdac3^Cdh5KO^* or *Hdac3^Lyve1KO^* hearts (Supplemental Figure 2, A and B). Among class I HDACs, murine embryos lacking Hdac1 or Hdac2 in the endothelial cells (*Hdac1^TekKO^* or *Hdac2^TekKO^*) appeared normal, with complete segregation of blood and lymphatic vasculature (Supplemental Figure 3, A and B). To determine whether ubiquitously expressed Hdac3 functions within platelets to maintain separation of the venous and lymphatic vasculature during development, we deleted Hdac3 in the platelets using *PF4-iCre* (*Hdac3^PF4KO^*) (53). *Hdac3^PF4KO^* neonatal mice were viable, appeared normal, and displayed complete blood-lymph separation (Supplemental Figure 4, A–S). Together, these results suggest that Hdac3 functions in LECs to regulate separation of the blood and lymphatic systems during development.

### Hdac3 regulates lymphovenous valve development.

**Figure 2:**
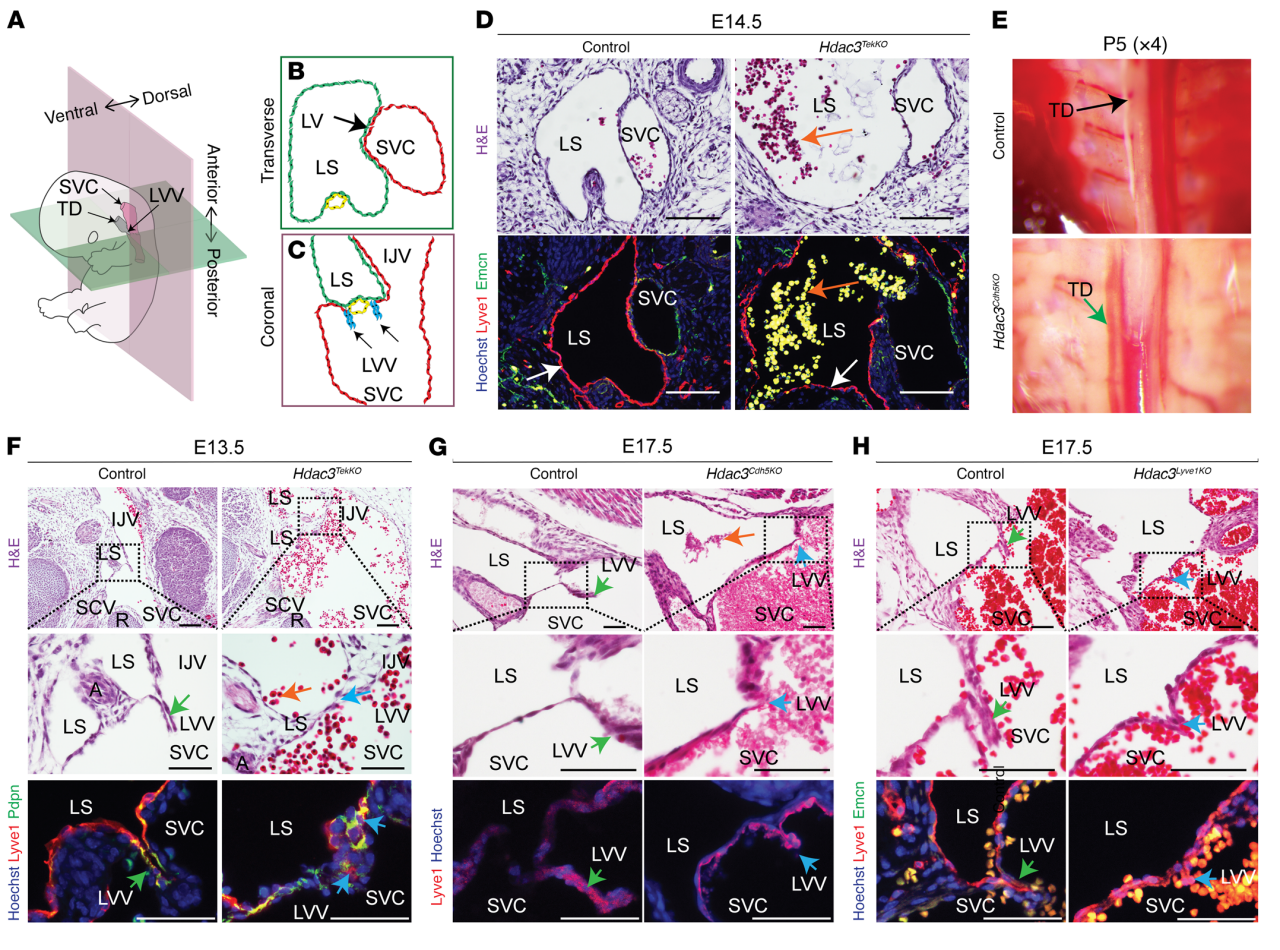
Hdac3 is an important regulator of lymphovenous valve development. (**A**–**C**) Schematic model depicting normal anatomy of a developing murine lymphovenous valve (**A**, black arrow) in transverse (**B**) and coronal (**C**) planes. (**D**) Transverse sections of E14.5 *Hdac3^TekKO^* embryos revealed blood-filled lymph sacs (orange arrows) lined by lymphatic (Lyve1 immunostaining [red], white arrows), but not venous (Emcn immunostaining, green), endothelial cells compared with that seen in controls. (**E**) Dissected P5 *Hdac3^Cdh5KO^* mice had a blood-filled thoracic duct (green arrow) compared with a chyle-filled thoracic duct in control mice (black arrow). (**F**) H&E-stained coronal sections of an E13.5 *Hdac3^TekKO^* embryo revealed a blood-filled lymph sac (orange arrow) and disrupted morphology of the lymphovenous valves (green arrows) compared with controls (yellow arrows). Immunofluorescence staining for podoplanin (Pdpn) (green) and Lyve1 (red) showed overlapping expression (yellow) in E13.5 LVVs. Orange arrow indicates a blood-filled lymph sac. (**G** and **H**) H&E-stained coronal sections of E17.5 *Hdac3^Cdh5KO^* (**G**) and *Hdac3^Lyve1KO^* (**H**) embryos revealed disrupted morphology of the lymphovenous valves (blue arrows) compared with controls (green arrows). Orange arrow shows a blood-filled lymph sac. Lyve1 (red) was expressed in E17.5 murine lymphovenous valves (**G** and **H**). Emcn (green, venous marker) was used as a negative control for lymphovenous valves (**H**). IJV, internal jugular vein; LS, lymph sac; LVV, lymphovenous valve; SVC, superior vena cava; TD, thoracic duct. Scale bars: 100 μm and 50 μm (**F**, bottom panels, **G**, and **H**). See also Supplemental Figure 5 and Supplemental Table 5.

Bicuspid lymphovenous valves, located at the thoracic duct–subclavian vein junction and right lymphatic duct–subclavian vein junction, maintain the separation between the high-pressure vascular system and the low-pressure lymphatic system (3–5). To investigate blood pooling in the jugular region of the embryos lacking endothelial Hdac3 (Figure 1B), we examined the morphology of developing lymphovenous valves and lymph sac in transverse and coronal sections (Figure 2, A–C). Embryos lacking endothelial Hdac3 displayed abnormal blood-filled lymph sacs at various developmental stages (Figure 2, D, F, and G, and Supplemental Figure 5). Endothelial cells lining blood-filled lymph sacs in Hdac3-null embryos revealed similar expression levels of Lyve1 or Vegfr3 (Figure 2D and Supplemental Figure 5). Consistent with this observation, the thoracic duct in *Hdac3^Cdh5KO^* neonates showed infiltration of blood into the normally lymph-filled duct, suggesting a functional lymphovenous valve defect (Figure 2E). Indeed, *Hdac3^TekKO^*, *Hdac3^Cdh5KO^*, and *Hdac3^Lyve1KO^* embryos displayed shortened lymphovenous valve leaflets with defective perpendicular alignment to the flow direction (Figure 2, F–H).

### Hdac3 regulates proper lymphatic transport and mesenteric lymphatic valve maturation.

**Figure 3:**
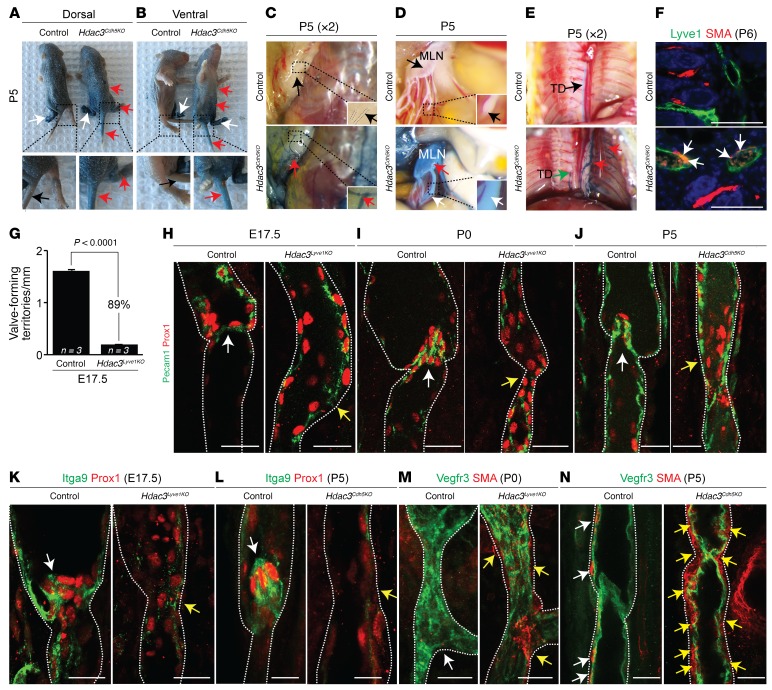
Hdac3 deficiency causes impaired lymphatic drainage and anomalous lymphatic valve development in mesenteric lymphatic vessels. (**A** and **B**) Evans blue dye injection into the left hind paw (**A** and **B**, white arrows) of P5 *Hdac3^Cdh5KO^* mice showed reflux (retrograde flow) into the tail, right hind limb, and abdomen (**A** and **B**, red arrows) compared with an absence of reflux in the control mice (black arrows). Original magnification ×2.5. (**C** and **D**) P5 *Hdac3^Cdh5KO^* mice showed Evans blue dye reflux (red arrows) into dermal lymph vessels and mesenteric lymph nodes compared with control mice (dotted line, black arrows). Original magnification ×3. (**E**) Control P5 mice showed normal unidirectional cephalad drainage restricted to the thoracic duct (black arrow). *Hdac3^Cdh5KO^* mice exhibited reflux into the intercostal lymphatics lateral to the thoracic duct in the thoracoepigastric region (red arrows) and reduced drainage into the thoracic duct (green arrow). (**F**) Coimmunofluorescence staining for Lyve1 (green) and smooth muscle actin (SMA) (red) showed abnormal smooth muscle recruitment (white arrows) to blood-filled dermal lymphatic capillaries in P6 *Hdac3^Cdh5KO^* mice compared with controls. (**G**) Quantitation of lymphatic valve territories in E17.5 *Hdac3^Lyve1KO^* mesenteric lymphatic vessels. The P value was determined by unpaired Student’s *t* test. (**H**–**J**) Whole-mount coimmunofluorescence staining of *Hdac3^Lyve1KO^* (**H** and **I**) and *Hdac3^Cdh5KO^* (**J**) mesenteric lymphatic vessels showed a lack of Prox1-expressing (red) lymphatic valves in Pecam1^+^ (green) lymphatic vessels (yellow arrows) compared with controls (white arrows). (**K** and **L**) Whole-mount coimmunofluorescence staining of *Hdac3^Lyve1KO^* (**K**) and *Hdac3^Cdh5KO^* (**L**) mesenteric lymphatic vessels showed a lack of Itga9-expressing (green) lymphatic valves in Prox1^+^ (red) lymphatic vessels (yellow arrows) compared with controls (white arrows). (**M** and **N**) Whole-mount coimmunofluorescence staining of *Hdac3^Lyve1KO^* (**M**) and *Hdac3^Cdh5KO^* (**N**) mesenteric lymphatic vessels showed excessive SMA (red) coverage (yellow arrows) compared with controls (white arrows). Vegfr3 (green) was used as a lymphatic vessel marker. Data represent the mean ± SEM and are representative of 3 independent experiments. Sub-stacks of *Z*-stack images are presented in **H**–**N**. Scale bars: 100 μm (**F**) and 1 μm (**H**–**N**). See also Supplemental Figure 6.

Dysfunctional intraluminal lymphatic valves in collecting lymphatic vessels impede the ability of the lymphatic system to effectively transport lymph, leading to lymphedema (6, 7). To study the lymph transport in *Hdac3^Cdh5KO^* neonates, we determined retrograde lymphatic flow reflux using Evans blue dye (Figure 3, A–E). *Hdac3^Cdh5KO^* neonates had abnormal retrograde flow of Evans blue dye into the tail, right hind limb, abdomen, dermal lymphatic vessels, mesenteric lymphatic vessels, and mesenteric lymph nodes (Figure 3, A–D). We also observed Evans blue dye in intercostal lymphatics lateral to the thoracic duct in the thoracoepigastric region, coupled with reduced contrast drainage into the thoracic duct in *Hdac3^Cdh5KO^* neonates compared with the control (Figure 3E), suggesting a severe impairment of lymph transport. Consistent with this observation, Lyve1^+^ dermal lymphatic capillaries revealed anomalous recruitment of smooth muscle cells in P6 *Hdac3^Cdh5KO^* neonates (Figure 3F). Additionally, Hdac3-deficient mesenteric lymphatic vessels showed immature and reduced numbers of lymphatic valves at various stages of development (Figure 3, G–N, and Supplemental Figure 6, A–D). Prox1 and Pecam1 whole-mount immunofluorescence costaining analyses revealed fewer and disorganized Prox1^Hi^ LEC clusters in *Hdac3^Cdh5KO^* and *Hdac3^Lyve1KO^* lymphatic vessels (Figure 3, H–J, and Supplemental Figure 6, A–D). *Hdac3^Cdh5KO^* and *Hdac3^Lyve1KO^* lymphatic vessels showed reduced integrin-α9 expression within lymphatic valve sites (Figure 3, K and L). In addition, Hdac3-deficient lymphatic collecting vessels exhibited excessive smooth muscle cell coverage compared with controls (Figure 3, M and N).

### Hdac3 regulates Gata2 expression in developing lymphovenous and lymphatic valves.

**Figure 4:**
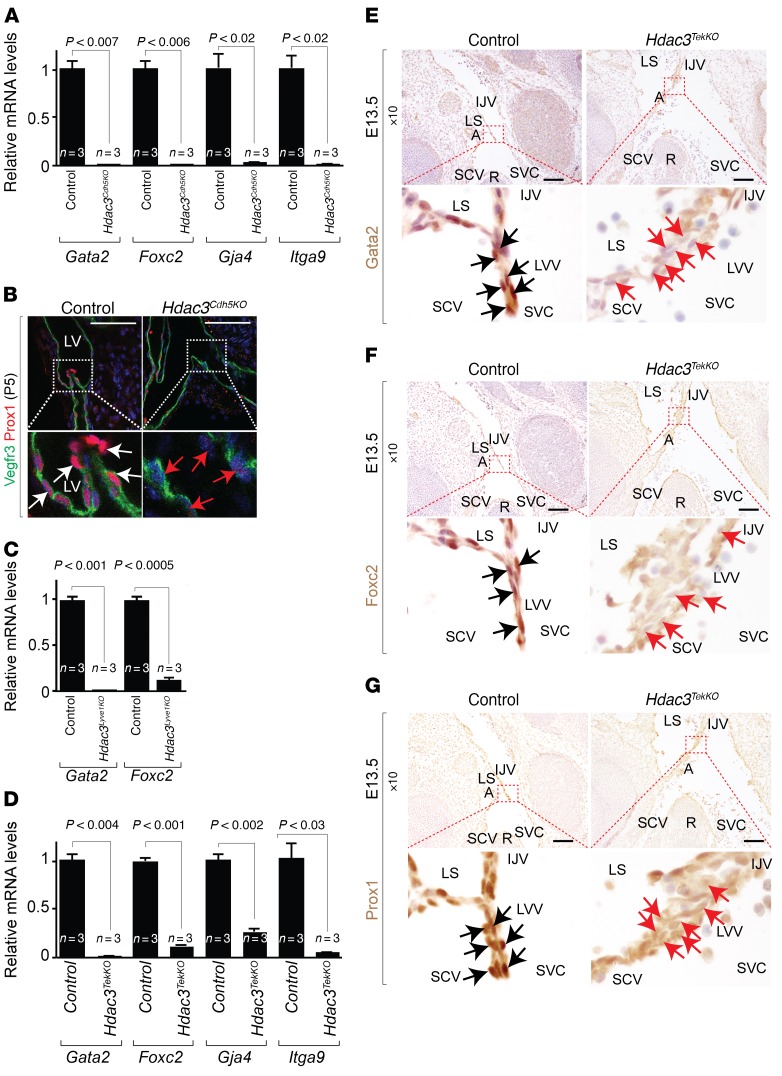
Hdac3 regulates Gata2 and its target gene expression in developing lymphatic valves and lymphovenous valves. (**A**) Transcripts for *Gata2, Foxc2, Gja4*, and *Itga9* were detected by ChIP-qPCR in control and *Hdac3^Cdh5KO^* mesenteric lymphatic vessels dissected from P5 mice. (**B**) Whole-mount immunofluorescence staining of *Hdac3^Cdh5KO^* mesenteric lymphatic vessels identified reduced Prox1 (red) expression in Vegfr3^+^ (red) LECs (red arrows) compared with controls (white arrows). (**C**) Transcripts for *Gata2* and *Foxc2* were detected by ChIP-qPCR in control and *Hdac3^Lyve1KO^* mesenteric lymphatic vessels dissected from P0 mice. (**D**) Transcripts for *Gata2, Foxc2, Gja4,* and *Itga9* were detected by ChIP-qPCR in laser-captured control and *Hdac3^TekKO^* lymphovenous valves from E13.5 embryos. (**E**–**G**) Immunostaining of E13.5 *Hdac3^TekKO^* lymphovenous valves revealed reduced Gata2 (**E**, red arrows), Foxc2 (**F**, red arrows), and Prox1 (**G**, red arrows) expression compared with controls (black arrows). LV, lymphatic valve; R, rib. Data represent the mean ± SEM and are representative of 3 independent experiments. *P* values were determined by Student’s *t* test. Scale bars: 500 μm. See also Supplemental Figure 7.

Transcriptional analysis of mesenteric lymphatic vessels dissected from *Hdac3^Cdh5KO^* neonates revealed downregulation of genes required for lymphatic valve development (9, 10, 12), such as *Gata2*, *Foxc2*, *Gja4*, and *Itga9* (Figure 4A). *Hdac3^Cdh5KO^* lymphatic vessels had endothelial marker gene transcript expression levels similar to those observed in controls (Supplemental Figure 7A). Loss of Hdac3 in mesenteric lymphatic endothelium also resulted in reduced Prox1 expression (Figure 4B). Consistent with this observation, we found that *Hdac3^Lyve1KO^* lymphatic vessels had downregulated expression of *Gata2* and *Foxc2* (Figure 4C). Using laser-capture microdissection (LCMD), we collected lymphovenous valves from serial coronal sections of E13.5 murine embryos (Supplemental Figure 7, B–D). *Hdac3^TekKO^* lymphovenous valves revealed similar expression levels of endothelial marker gene transcripts (Supplemental Figure 7D), but downregulation of *Gata2*, *Foxc2*, *Gja4*, and *Itga9* transcripts, compared with expression levels in controls (Figure 4D). Consistent with this observation, IHC revealed reduced expression of Gata2, Foxc2, and Prox1 in the developing *Hdac3^TekKO^* lymphovenous valves (Figure 4, E–G).

### Hdac3 regulates OSS-mediated activation of the Gata2 intragenic enhancer.

**Figure 5:**
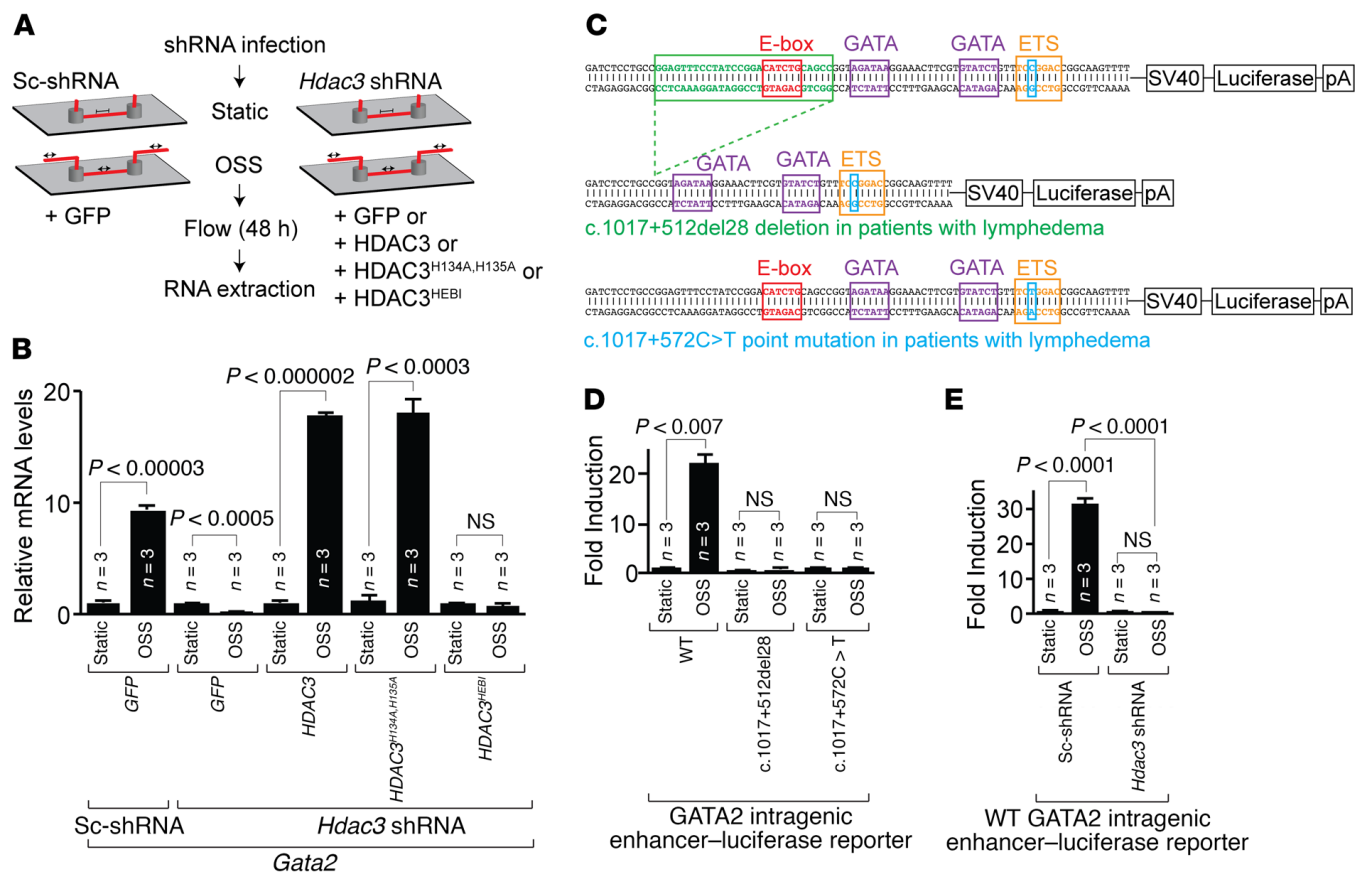
Hdac3 regulates oscillatory shear stress–mediated activation of the Gata2 intragenic enhancer. (**A**) Schematic of in vitro OSS assay. (**B**) Transcripts for *Gata2* were detected by ChIP-qPCR in scrambled shRNA– (Sc-shRNA) or *Hdac3* shRNA–infected LECs subjected to static or OSS conditions and coinfected with *GFP*, *HDAC3*, *HDAC3^H134A,H135A^*, or *HDAC3^HEBI^* lentiviruses. (**C**) Schematics of luciferase reporter vectors composed of WT (168 bp), c.1017+512del28 (28-bp deletion, green box, 140 bp), and the c.1017+572C>T point mutation (blue box) GATA2 intragenic enhancer. (**D**) A dual luciferase assay was performed in LECs transfected with WT, c.1017+512del28, or the c.1017+572C>T point mutation GATA2 intragenic enhancer luciferase reporter and subjected to static or OSS conditions. Induction is represented as a ratio of firefly to *Renilla* luciferase activity. (**E**) Scramble shRNA– or *Hdac3* shRNA–infected LECs, cotransfected with the WT GATA2 intragenic enhancer luciferase reporter, were subjected to static or OSS conditions. Induction is represented as a ratio of firefly to *Renilla* luciferase activity. Data represent the mean ± SEM and are representative of 3 independent experiments. *P* values were determined by Student’s *t* test (**B** and **D**) or by 1-way ANOVA with Sidak’s multiple comparisons test (**E**). See also Supplemental Figures 8 and 9.

Lymph flow and OSS upregulate genes required for lymphatic valve development including Gata2 (8, 9, 12). Gata2, an important transcription factor for blood-lymph separation and development of lymphovenous and lymphatic valves, is an upstream transcriptional regulator of Foxc2, Prox1, Gja4, and Itga9 (9, 10, 13, 14). In addition, the Gata2 intragenic enhancer is both sufficient and necessary to drive Gata2 expression within LECs (14, 23). Ablation of this Gata2 intragenic enhancer in mice is sufficient to reduce Gata2 expression and phenocopy the endothelial knockout of Gata2 (24). Hence, we examined the mechanism by which Hdac3 regulates Gata2 expression. To investigate whether Hdac3 regulates Gata2 expression in response to shear stress, we analyzed LECs cultured under static or OSS conditions (Figure 5A). LECs showed upregulation of *Gata2*, *Foxc2*, and *Gja4* in response to OSS, and Hdac3 knockdown abolished this effect (Figure 5B and Supplemental Figure 8A). Catalytically inactive enzymes are frequently present in most enzyme families including HDACs (54). The biological functions of these pseudoenzymes remain largely unknown. To determine whether deacetylase activity or chromatin recruitment of Hdac3 is required to regulate Gata2 expression in response to OSS, we overexpressed 2 different mutant forms of human HDAC3 in Hdac3-deficient LECs. The first mutant, HDAC3^HEBI^, exhibits no enzymatic activity or chromatin recruitment, while the second mutant, HDAC3^H134A,^ ^H135A^, lacks enzymatic activity but has preserved chromatin recruitment (40, 55). Flag-tagged WT and mutant HDAC3 plasmids showed similar levels of expression within LECs (Supplemental Figure 8B). Expression of either WT Hdac3 or HDAC3^H134A,^ ^H135A^ rescued Gata2 expression, whereas HDAC3^HEBI^ expression failed to do so (Figure 5B). These data indicate that Hdac3 functions in a chromatin-dependent, but deacetylase-independent, manner to regulate Gata2 expression within LECs in response to OSS.

The intron 5 168-bp GATA2 intragenic enhancer region is highly conserved (Supplemental Figure 9). Importantly, both the c.1017+512del28 deletion region and the c.1017+572C>T mutations encompass the evolutionarily conserved E-box–GATA composite element and ETS motif, respectively (Supplemental Figure 9). To test the transcriptional activity of this Gata2 intragenic enhancer in response to OSS, we generated luciferase reporter vectors containing the WT murine Gata2 intragenic enhancer, the c.1017+512del28 variant lacking its E-box element, and the c.1017+572C>T variant abolishing the ETS motif (Figure 5C). OSS increased transcriptional activity of the WT Gata2 intragenic enhancer within LECs (Figure 5D). In contrast, OSS failed to induce either mutant reporter, suggesting that the E-box–GATA composite element and the ETS motif are both required for transcriptional activation of the GATA2 intragenic enhancer in response to OSS (Figure 5D). Importantly, loss of Hdac3 abolished OSS-dependent transcriptional activation of the Gata2 intragenic enhancer reporter within LECs (Figure 5E).

### Tal1, Gata2, and Ets1/2 recruit Hdac3 to the Gata2 intragenic enhancer in a shear stress–dependent manner.

**Figure 6:**
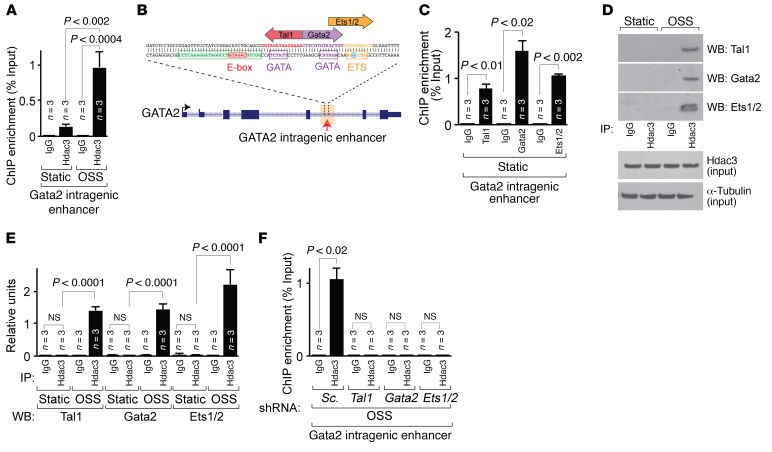
Tal1, Gata2, and Ets1/2 recruit Hdac3 to the Gata2 intragenic enhancer in response to lymphatic shear stress. (**A**) ChIP-qPCR analysis of Hdac3 recruitment to the Gata2 intragenic enhancer was performed in LECs subjected to static conditions or OSS. (**B**) Schematic model depicting TRANSFAC analysis of ENCODE ChIP-seq data sets at the E-box–GATA–ETS element of the Gata2 intragenic enhancer. (**C**) ChIP–qPCR analysis of Tal1, Gata2, and Ets1/2 recruitment to the Gata2 intragenic enhancer was performed in LECs. (**D** and **E**) Total lysates from pooled LECs subjected to static or OSS conditions were immunoprecipitated by IgG or Hdac3 antibody, and Western blot analysis was performed using Tal1, Gata2, or Ets1/2 antibody (**D**). Total Hdac3 and α-tubulin levels are shown as an input control (**D**). Tal1, Gata2, and Ets1/2 expression was quantified and normalized to total input using ImageJ software (**E**). (**F**) ChIP–qPCR analysis of Hdac3 recruitment to the Gata2 intragenic enhancer in scrambled shRNA–, *Tal1* shRNA–, *Gata2* shRNA–, or *Ets1/2* shRNA–infected LECs subjected to OSS (*n* = 3). Data represent the mean ± SEM and are representative of 3 independent experiments. *P* values were determined by Student’s *t* test (**C** and **F**) or by 1-way ANOVA using Sidak’s multiple comparisons test (**A** and **E**). WB, Western blotting. See also Supplemental Figure 10.

To investigate the mechanisms by which Hdac3 regulates the Gata2 intragenic enhancer, we determined enrichment of Hdac3 at the Gata2 intragenic enhancer region in LECs cultured under static or OSS conditions. Hdac3 occupancy at the Gata2 intragenic enhancer was increased under OSS compared with that observed under static conditions, suggesting that lymphatic flow regulates the recruitment of Hdac3 to chromatin (Figure 6A). TRANSFAC (TRANScription FACtor database; http://genexplain.com/transfac/) analysis of ENCODE (Encyclopedia of DNA Elements; https://www.encodeproject.org/) ChIP-sequencing (ChIP-seq) data sets suggested a composite occupancy of the transcription factors Tal1, Gata2, and Ets1/2 at the E-box–GATA–ETS element of the Gata2 intragenic enhancer (Figure 6B). ChIP–quantitative PCR (ChIP-qPCR) analysis confirmed enrichment of Tal1, Gata2, and Ets1/2 at the Gata2 intragenic enhancer region in LECs under static conditions (Figure 6C). To test the physical interaction of Hdac3 with Tal1, Gata2, or Ets1/2, we performed immunoprecipitation of endogenous Hdac3 protein from LECs cultured under static or OSS conditions, followed by immunoblotting for Tal1, Gata2, or Ets1/2. We found that OSS induced a physical interaction between Hdac3 and Tal1, Gata2, or Ets1/2 in LECs (Figure 6, D and E). Notably, total expression of Hdac3 protein and *Tal1* and *Ets1* transcripts remained unchanged in LECs in response to OSS (Figure 6D and Supplemental Figure 10A). Loss of Tal1, Gata2, or Ets1/2 abolished OSS-dependent recruitment of Hdac3 to the Gata2 intragenic enhancer in LECs (Figure 6F and Supplemental Figure 10B). LECs expressing *Tal1*, *Gata2*, or *Ets1/2* shRNAs showed similar expression levels of *Hdac3* transcripts (Supplemental Figure 10C).

### Hdac3 recruits EP300 to the Gata2 intragenic enhancer to promote Gata2 transcription in response to OSS.

**Figure 7:**
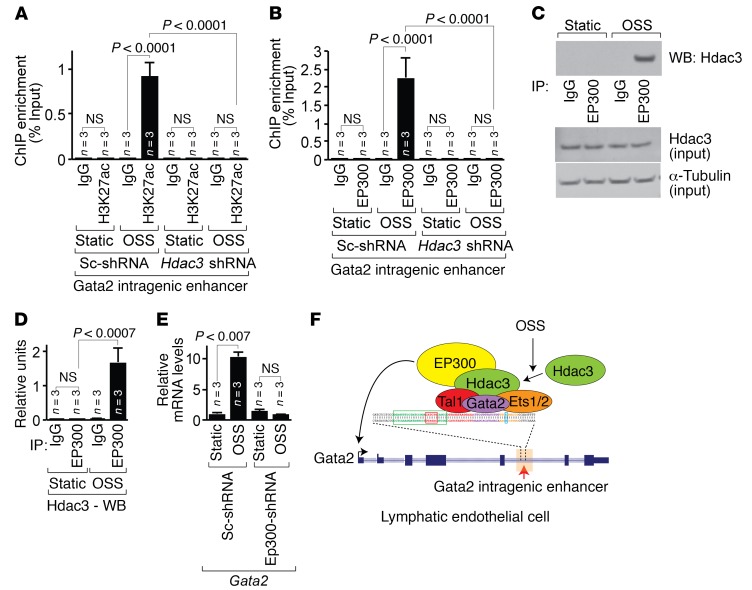
Hdac3 recruits EP300 to the Gata2 intragenic enhancer in response to lymphatic shear stress. (**A** and **B**) ChIP–qPCR analysis of H3K27 acetylation (**A**) and EP300 recruitment (**B**) to the Gata2 intragenic enhancer in scrambled shRNA– or *Hdac3* shRNA–infected LECs subjected to static or OSS conditions. (**C** and **D**) Total lysates from pooled LECs subjected to static or OSS conditions were immunoprecipitated by IgG or EP300 antibody, and Western blot analysis was performed using Hdac3 antibody (**C**). Total Hdac3 and α-tubulin levels are shown as input controls. Hdac3 expression was quantified and normalized to total input using ImageJ software (*n* = 3) (**D**). (**E**) Transcripts for *Gata2* were detected by ChIP-qPCR in scrambled shRNA– or *EP300*-shRNA–infected LECs subjected to static or OSS conditions. (**F**) In response to OSS, the transcription factors Tal1, Gata2, and Ets1/2 recruit Hdac3 to the Gata2 intragenic enhancer, which in turn recruits EP300 to promote Gata2 expression. In LECs lacking Hdac3, OSS failed to promote EP300 recruitment to the Gata2 intragenic enhancer, histone H3 Lys-27 acetylation, and, thereby, Gata2 expression. Data represent the mean ± SEM and are representative of 3 independent experiments. *P* values were determined by Student’s *t* test (**E**) or by 1-way ANOVA with Sidak’s multiple comparisons test (**A**, **B**, and **D**). See also Supplemental Figure 10.

To investigate how Hdac3 regulates the activation of the Gata2 intragenic enhancer in response to shear stress, we determined the enrichment of H3K27ac and EP300 at the Gata2 intragenic enhancer in WT and Hdac3-deficient LECs cultured under static or OSS conditions. LECs showed enrichment of H3K27ac and EP300 in response to OSS, and this enrichment was abolished by Hdac3 knockdown (Figure 7, A and B, and Supplemental Figure 10B). We observed that *Ep300* transcript expression levels remained unchanged in LECs in response to OSS (Supplemental Figure 10A). To test the physical interaction of Hdac3 and EP300, we performed immunoprecipitation of endogenous EP300 protein from LECs cultured under static or OSS conditions, followed by immunoblotting for Hdac3. OSS induced physical interaction between Hdac3 and EP300 in LECs (Figure 7, C and D). Taken together, these results suggest that Hdac3 recruits EP300 to the Gata2 intragenic enhancer in response to OSS. By contrast, EP300-deficient LECs failed to demonstrate transcriptional activation of Gata2 in response to OSS (Figure 7E and Supplemental Figure 10B). LECs expressing *Tal1*, *Gata2*, or *Ets1/2* shRNAs showed similar expression levels of *Ep300* transcripts (Supplemental Figure 10C). These data suggest that EP300 recruitment to the Gata2 intragenic enhancer is important for lymphatic flow–dependent Gata2 expression (Figure 7F).

## Discussion

This study reveals a unique, unexpected, and nonredundant role of Hdac3 as a key regulator of blood-lymph separation during development. Our results demonstrate that endothelial inactivation of ubiquitously expressed Hdac3, but not Hdac1 or Hdac2, in mice causes embryonic lethality, failure of blood-lymph separation, lymphedema, and defects in both lymphatic and lymphovenous valves. Despite the sequence homology among class I Hdacs, these results suggest a unique function of Hdac3 to mediate blood-lymph separation during development.

To our knowledge, this is the first report of a histone-modifying enzyme regulating separation of the blood and lymphatic vascular systems. Recent studies revealed that platelets and podoplanin (an LEC receptor) cooperate to prevent blood from entering the lymphatic system at the lymphovenous valve (4, 56–60). However, we found that ablation of Hdac3 in platelets caused no defects in blood-lymph separation (Supplemental Figure 4, A–S), suggesting that Hdac3 functions specifically within LECs. Here, we show that Hdac3 ablation in LECs prior to lymphovenous valve formation (*Hdac3^TekKO^*) resulted in a failure of blood-lymph separation at E12.5 and lethality as early as E13.5 (Supplemental Figure 5 and Supplemental Tables 1 and 2). Similarly, complete loss of Hdac3 in LECs after lymphovenous valve formation (*Hdac3^Cdh5KO^* or *Hdac3^Lyve1KO^*) led to a failure of blood-lymph separation soon after birth (Figure 1, B, C, and E–I), followed by complete neonatal lethality (Supplemental Tables 3 and 4). These alternative genetic approaches reveal that the timing of lethality in mice lacking LEC Hdac3 coincides with lymphovenous valve dysfunction and failure of blood-lymph separation. However, Hdac3-deficient lymphovenous valves and lymphatic vessels showed similar podoplanin expression levels compared with levels detected in controls (Figure 2F and Supplemental Figure 7, A and D).

These results raise an interesting question: How does ubiquitously expressed Hdac3 function in a LEC-specific manner during development? Lacking intrinsic DNA-binding capacity, Hdac3 must be recruited to the chromatin via its interaction with multiple protein nuclear complexes, transcription factors, and cofactors (32). Emerging data from genome-wide mapping reveal that approximately 60% of Hdac3 occupies intragenic and intergenic regions including those marked by H3K27ac (43–47). This distribution suggests enhancer-specific functions for Hdac3. Enhancers recruit a multitude of protein complexes to orchestrate gene expression in a temporal, spatial, or cell type–specific manner (28). The GATA2 intragenic enhancer is both sufficient and necessary to drive GATA2 expression in LECs (14, 23). Our findings support a model in which Hdac3 functions in concert with EP300 to activate, rather than repress, the Gata2 intragenic enhancer and thereby establish a specific transcriptional program for LECs. This model challenges long-held assumptions that HDACs replace HATs to promote both histone deacetylation and repression of transcription. Taken together, our findings warrant further investigation of the extent, if any, to which the intragenic and intergenic occupancy of HDACs contributes to the regulation of enhancer functions that modulate gene expression.

Our finding that HDAC3 contributes to the function of the GATA2 intragenic enhancer dovetails with recent GWAS revealing that the majority of genetic variants or mutations associated with human diseases map to the noncoding elements in the genome (61). A substantial fraction of such mutations are thought to disrupt enhancer elements to alter gene expression and cause disease; however, only a few examples have been identified (62). Consistent with this notion, patients with c.1017+512del28 deletion or a c.1017+572C>T point mutation within the evolutionarily conserved GATA2 intragenic enhancer (divergence ~350 million years ago) have lymphedema and significantly reduced GATA2 expression (20–22, 63). The present study takes this scenario one step further by revealing that Hdac3 functions as an important epigenetic switch to promote enhanceosome complex formation at a deeply conserved Gata2 intragenic enhancer element in response to OSS. Disruption of the E-box or ETS motif nullifies Hdac3-mediated induction of Gata2 expression, suggesting that perturbation of these regions disrupts the assembly of the Tal1-Gata2-Ets1/2-Hdac3-Ep300 enhanceosome in developing lymphatic endothelium. These results suggest a mechanism by which c.107+512del28 or c.107+572C>T mutations cause lymphedema. This model supports the hypothesis that enhanceosomes function as a transcription activation switch for developmentally important transcription factors in response to intrinsic or extrinsic signaling cues (64, 65). While we do not yet fully understand the nature of the enhancer elements in the developing lymphatic system, these studies broaden the role of enhanceosomes at deeply conserved enhancer elements in lymphatic valve development.

Here, we highlight the integrated nature of extracellular forces and epigenetic signatures at the enhancer elements. Our data show that OSS promotes assembly of the enhanceosome complex containing the transcription factors Gata2, Tal1, Ets1/2 and the histone-modifying enzymes Hdac3 and EP300 at the Gata2 intragenic enhancer–specific E-box–GATA–ETS composite element. Concentrated clusters of transcription factor recognition motifs within the composite element facilitate direct cooperative binding of multiple transcription factors prior to enhancer activation (64). Such “priming” of enhancer elements allows additional recruitment of both cofactors and effectors of signal transduction pathways to integrate intrinsic and extrinsic signaling cues during key stages of development (27). We propose that cooperative interactions among Gata2, Tal1, and Ets1/2 at the E-box–GATA–ETS composite element lead to priming of the Gata2 intragenic enhancer. OSS-mediated mechanotransduction promotes recruitment of Hdac3 and thereby EP300 at the primed Gata2 intragenic enhancer to modify the epigenetic signature, which in turn rapidly activates Gata2 transcription (Figure 7F). In line with this concept, developing LECs subjected to OSS exhibit robust upregulation of Gata2, which is important for lymphatic valve development (9, 10). In addition, murine embryos lacking endothelial Tal1 display edema, blood-filled superficial vessels, and complete lethality at E14.5 (66). Global loss of both Ets1 and Ets2 in murine embryos also causes edema, blood-filled dilated superficial vessels, pooling of blood in the jugular region, and complete lethality at E15.5 (67). These phenotypes appear very similar to those in murine embryos lacking endothelial Hdac3. Hence, our data warrant a reexamination of Tal1-knockout and Ets1/2-knockout phenotypes to determine whether Tal1 and Ets1/2 regulate blood-lymph separation and lymphatic valve development.

While the transcriptional regulators of Gata2 are defined, the epigenetic modifiers remain unknown. Our data identify Hdac3 as a positive transcriptional regulator of *Gata2*. Consistent with this notion, embryos lacking endothelial Hdac3 (our observations) or Gata2 (10, 56) show highly similar phenotypes. For instance, endothelial ablation of Hdac3 causes shortened lymphovenous valve leaflets with defective perpendicular alignment to the flow direction, similar to those seen in murine embryos lacking endothelial Gata2 (56). Loss of endothelial Hdac3 or Gata2 also causes reduced expression of Foxc2 and Prox1 within developing murine lymphovenous valves (10, 56). In addition, embryos lacking lymphatic Hdac3 or Gata2 have dysplastic mesenteric lymphatic vessels with fewer and disorganized valve-forming territories (10). Furthermore, Hdac3 or Gata2 ablation in blood endothelial cells causes failure of blood-lymph separation in murine embryos between E12.5 and E13.5 (10, 56). Interestingly, Prox1-CreERT2–mediated inducible Gata2 ablation in lymphatic vasculature at E10.5, prior to lymphovenous valve formation, recapitulates this phenotype at E13.5 (10). In contrast, Lyve1-Cre–mediated Hdac3 deletion in lymphatic vasculature causes blood-lymph separation failure between E17.5 and P0 (Figure 1, C and I, and Supplemental Figure 1G). Pan-endothelial *Cre*-expressing mice (*Tek-Cre* mice) show recombinase activity in LEC precursors at E10.5, prior to lymphovenous valve formation (68). However, Lyve1-Cre–expressing embryos reveal fully penetrant recombinase activity within lymphovenous valves at E14.5, after lymphovenous valve formation (69). These alternative genetic approaches reveal that failure of blood-lymph separation due to lymphovenous valve defects corresponds with the timing of endothelial Hdac3 ablation in both *Lyve1-Cre* and *Tek-Cre* mice.

Overall, our study suggests that enhancer-specific epigenetic signatures are functional components of mechano-induced transcriptional responses during lymphatic valve development. Future studies will define the precise mechanotransduction and mechanosensory pathways that integrate extracellular signaling at enhancers during lymphatic vascular development.

## Methods

### Mice.

*Tek-Cre* (51), *Cdh5-Cre* (50), *Lyve1-Cre* (52), *Pf4-iCre* (codon-improved *Cre* recombinase) (53), and *Gt(ROSA)26Sor* (70) reporter mice were obtained from The Jackson Laboratory. *Hdac1^fl/fl^* (71), *Hdac2^fl/fl^* (72), and *Hdac3^fl/fl^* (47) mice have been previously described.

### Antibodies and reagents.

Detailed antibody information is provided in Supplemental Table 6. Biotinylated secondary antibodies, VECTASHIELD mounting medium, the VECTASTAIN Elite ABC Kit, and the DAB Peroxidase Substrate Kit were purchased from Vector Laboratories. Harris modified hematoxylin, eosin Y, ethanol, methanol, chloroform, glacial acetic acid, xylenes, paraformaldehyde, paraffin, potassium ferricyanide, potassium ferrocyanide, and deoxycholic acid were purchased from Thermo Fisher Scientific. X-Gal was purchased from Five Prime Therapeutics. Linear polyethylenamine (PEI) was purchased from Polysciences. The RNeasy Mini Kit and GST bead slurry were purchased from QIAGEN. Power SYBR Green PCR Master Mix, a Superscript First Strand Synthesis Kit, a TOPO-TA Cloning Kit, DMEM high-glucose with NA pyruvate, penicillin-streptomycin, horse serum, a CellsDirect One-Step qRT-PCR Kit, insulin-transferrin-selenium (ITS), epoxy M-450 dynabeads, and TRIzol were purchased from Life Technologies (Thermo Fisher Scientific). iScript Reverse Transcription Supermix was purchased from Bio-Rad. Passive lysis buffer and a dual-luciferase reporter assay kit were purchased from Promega. BALB/c murine primary LECs, basal medium, and complete mouse endothelial cell medium were purchased from Cell Biologics. FBS, donkey serum, gelatin, rabbit serum, Evans blue dye, protease inhibitor mixture, and magnetic anti-Flag beads were purchased from MilliporeSigma. Agarose IgG and IgA bead slurry were purchased from Santa Cruz Biotechnology and Life Technologies (Thermo Fisher Scientific). μ-Slide VI 0.4 Luer was purchased from Ibidi. The EZ-ChIP Assay Kit was purchased from MilliporeSigma. The RecoverAll Total Nucleic Acid Isolation Kit was purchased from Thermo Fisher Scientific. The CellAmp Whole Transcriptome Amplification Kit and the Takara DNA Ligation Kit were purchased from Takara. Membrane slides and isolation caps were purchased from Molecular Machines & Industries.

### Plasmids.

WT, c.1017+512del28, and c.1017+572C>T Gata2 intragenic enhancer fragments with flanking restriction enzyme sites were purchased as MiniGenes cloned into a pIDTSMART-AMP vector from Integrated DNA Technologies. The enhancer fragments were digested with MluI and XhoI, gel purified, and cloned into the pGL3-Promoter Luciferase Vector (Promega). All plasmids were verified by restriction analyses and sequencing (Eurofins MWG Operon). Lentiviral plasmids expressing *Hdac3* shRNA, *EP300* shRNA, *Tal1* shRNA, *Ets1/2* shRNA, *Gata2* shRNA, and scrambled shRNA controls were obtained from the University of Massachusetts shRNA Core Facility. Plasmids expressing human *GFP*, *HDAC3*, *HDAC3^HEBI^*, and *HDAC3^H134A,^^H135A^* have been previously described (40, 55).

### Imaging.

Images of dissected embryos, mice, and tissue sample were captured using a Leica MZ10 F fluorescence stereomicroscope equipped with a ×0.7 C-mount, Achromat 1.0 × 90 mm objective, a SOLA light engine, a DS-Fi1 color camera (Nikon), and NIS-Elements Basic Research software (Nikon). Stained section images were captured using a Nikon Eclipse 80i microscope equipped with CFI Plan Fluor ×4/×10/×20/×40 objective lenses, a SOLA light engine (Lumencor), a DS-Fi1 color camera, and NIS-Elements Basic Research software. Whole-mount immunostained images were captured using a Zeiss LSM710 confocal scanning microscope equipped with a W Plan-Apochromat ×20/1.0 DIC D = 0.17 M27 70-mm objective lens as previously described (73). Whole-mount immunostained mesenteric vessels were flat mounted onto histobond glass slides using VECTASHIELD mounting medium for immunofluorescence and imaged using a Nikon Eclipse 80i microscope or a Leica TCS SP5 II Laser Scanning Confocal microscope. Alexa Fluor 488 and 568 were simultaneously excited at 488 nm and 561 nm with confocal lasers, respectively. Emissions were split by an MBS 488/561/633 beam splitter and captured with 2 detection ranges (ch1: 493–536 nm, ch2: 576–685 nm). For nuclear staining, Hoechst was excited using a Chameleon Ti:Sapphire pulse laser (755 nm) (Coherent Inc.) and was emission detected at 387 to 486 nm. Image stacks of vertical projections were assembled using ImageJ software (NIH).

### Histology.

Embryos and tissues samples were fixed in 2% paraformaldehyde at 4°C overnight, ethanol dehydrated, embedded in paraffin, and sectioned at 6- to 8-μm thickness using a Leica fully motorized rotary microtome.

### H&E staining.

Formalin-fixed, paraffin-embedded tissue sections were deparaffinized in xylene and rehydrated through an ethanol gradient, followed by 2-minute Harris modified hematoxylin and 30-second eosin-Y staining. Slides were dehydrated with ethanol, cleared with xylene, and mounted with VECTASHIELD mounting medium.

### In vitro OSS experiments.

Transiently transfected or infected murine primary LECs were seeded at confluence on μ-Slide VI 0.4 Luer, cultured for 24 hours, and subjected to OSS (4 dynes/cm^2^, 4 Hz) in a parallel-plate flow chamber system (Ibidi Pump System; Ibidi) or under static conditions for 48 hours.

### β-Gal staining.

Embryos were dissected and placed in ice-cold 1× PBS and then fixed in 4% paraformaldehyde for 1 hour at 4°C. Embryos were washed in 1× PBS for 30 minutes at room temperature and then incubated in β-gal staining solution (5 mM potassium ferricyanide, 5 mM potassium ferrocyanide, 2 mM MgCl2, 0.04% NP-40, 0.01% deoxycholate, and 0.1% X-gal substrate in 1× PBS) for 48 to 72 hours at 37°C in the dark. Embryos were then washed 3 times for 20 minutes each in 1× PBS at room temperature and fixed overnight in 4% paraformaldehyde.

### IHC.

IHC was performed as we previously described (40). Briefly, slides with sections were deparaffinized and immersed in sodium citrate buffer (10 mM sodium citrate, 0.05% Tween-20, pH 6) or R-buffer A (pH 6; Electron Microscopy Sciences) and placed in a 2100 Antigen Retriever (Aptum Biologics) for heat-induced antigen retrieval. IHC was conducted using a VECTASTAIN Elite ABC Kit and a DAB Peroxidase Substrate Kit (both from Vector Laboratories) according to the manufacturer’s guidelines. Sections were incubated with primary antibodies overnight at 4°C (Supplemental Table 6). Biotinylated universal pan-specific antibody (1:62.5) (horse anti-mouse/rabbit/goat IgG); biotinylated universal antibody (horse anti-mouse/rabbit IgG); or biotinylated rabbit anti-rat IgG antibody (1:62.5) (all from Vector Laboratories) were used as secondary antibodies for 1 hour at 25°C according to the manufacturer’s instructions. For counterstaining, slides were rinsed and then incubated with 30% hematoxylin for 30 seconds after DAB development. All slides were ethanol dehydrated, cleared with xylenes, and mounted with VECTASHIELD mounting medium. For immunofluorescence staining, after antigen retrieval, sections were blocked in 10% donkey serum and 0.3% Triton X-100 in PBS for 1 hour at room temperature. Sections were then washed in PBS and coincubated with primary antibodies in 10% donkey serum and PBS overnight at 4°C (Supplemental Table 6). Finally, slides were washed in PBS, incubated in Alexa Fluor 488– or 546/568–conjugated secondary antibodies (1:500) with Hoechst (1:1,000) for 1 hour at room temperature, rinsed in PBS, and mounted with VECTASHIELD mounting medium.

### Cell culture, transient transfection, lentiviral infection, and luciferase assay.

Murine primary LECs were maintained in complete mouse endothelial cell medium in a 37°C incubator with 5% CO_2_ according to the manufacturer’s protocol (Cell Biologics). All experiments were conducted using passage 3–6 LECs. HEK293T cells were maintained in DMEM with 10% FBS, 100 mg/ml penicillin, and 100 mM/ml streptomycin in a 37°C incubator with 5% CO_2_. Subconfluent HEK293T cells were transfected in 100-mm plates with 5 μg lentiviral plasmid expressing the relevant cDNA or shRNA, 5 μg pCMV-dR8.2, 2.5 μg pCMV-VSVG, and 5 μl PEI, in 10 ml of 2% FBS media. Media were changed to fresh FBS media (10 ml of 1%) 24 hours after transfection. Supernatant media were collected 24 hours later and filtered through a 40-μm cell strainer. Murine primary LECs were infected with fresh-filtered viral media supplemented with 10 μg/ml polybrene reagent. Murine primary LECs were transiently transfected using a PEI-based transfection protocol (39, 40, 74, 75). Each well of a 6-well plate containing subconfluent (~60% confluent) LECs was transfected with a total of 1 μg plasmid DNA, including 0.5 μg WT or c.1017+512del28, or the c.1017+572C>T Gata2 intragenic enhancer pGL3-promoter luciferase vector, with or without 0.5 μg *Hdac3* shRNA–expressing vector and 2 μl PEI (1 mg/ml in double-distilled H_2_O, pH 7.0), in 2 ml complete mouse endothelial cell medium. The PEI-DNA complex was incubated at room temperature for 20 minute before adding it to LECs in a drop-wise manner. The DNA amount was maintained constant using pcDNA3.1(-) DNA. LECs were trypsinized and reseeded to a μ-Slide VI 0.4 Luer (~100% confluence) 24 hours after transfection. Transiently transfected LECs were subjected to static or OSS conditions for 48 hours and lysed with passive lysis buffer. Lysates were analyzed using a dual luciferase reporter assay kit according to the manufacturer’s protocol (Promega). Luciferase activity was measured using a Berthold microplate reader according to the manufacturer’s guidelines.

### LCMD.

LCMD was performed according to the manufacturer’s protocol (Molecular Machines & Industries). Briefly, embryos were dissected at E13.5 in cold DEPC–treated 1× PBS, fixed in methacam (60% methanol, 30% chloroform, 10% glacial acetic acid) overnight at 4°C, ethanol dehydrated, embedded in paraffin, sectioned at 8-μm thickness, and mounted onto membrane slides. Tissue sections were deparaffinized in xylene and rehydrated through an ethanol gradient, followed by a 10-second hematoxylin staining. LCMD was performed on an MMI CellCut System equipped with a fixed UV laser with a higher pulse rate using MMI CellTools software, phase-contrast objectives, and an MMI CellCamera (all from Molecular Machines & Industries). Microdissected tissue was collected directly into an adhesive MMI isolation cap.

### ChIP.

ChIP experiments were performed as previously described (39). Briefly, primary LECs subjected to OSS or static conditions were pooled (total ~1 × 10^6^ cells), cross-linked for 15 minutes in cross-linking solution (1% formaldehyde, 1.5 mM ethylene glycol-bis succinimidyl succinate, 20 mM sodium butyrate, 10% FBS), and then quenched with 125 mM glycine solution for 5 minutes, followed by 2 washes with 1× PBS. Chromatin fragmentation was performed by sonication in ChIP SDS lysis buffer (50 mM Tris-HCl, pH 8.0, 10 mM EDTA, 1% SDS, 1× protease inhibitors) using the Branson Sonifier 250 (40% power amplitude, 120-second). Proteins were immunoprecipitated in ChIP dilution buffer (0.01% SDS, 1.1% Triton X-100, 1.2 mM EDTA, 16.7 mM Tris-HCl, pH 8.0, 167 mM NaCl, 20 mM sodium butyrate, 1× protease inhibitor) using IgG, Hdac3, Ep300, Tal1, Ets1/2, Gata2, or H3K27ac primary antibody. Specificity of the primary antibody at the relevant locus was determined using an IgG control antibody. Immunoprecipitated antibody-chromatin complexes were washed twice with low-salt wash buffer (20 mM Tris-HCl, pH 8.0, 150 mM NaCl, 2 mM EDTA, 0.1% SDS, and 1% Triton X-100), followed by 2 washes with lithium chloride wash buffer (10 mM Tris-HCl, pH 8.0, 250 mM LiCl, 1 mM EDTA, 1% deoxycholate, 1% Nonidet P-40) and TE buffer (10 mM Tris-HCl, pH 8.0, 1 mM EDTA). After removing the wash buffer, cross-linking was reversed at 65°C overnight in proteinase K buffer (20 mM Tris-HCl, pH 7.5, 5 mM EDTA, 50 mM NaCl, 1% SDS, 20 mM sodium butyrate, 50 μg/ml proteinase K). The following day, DNA was purified with phenol/chloroform/isoamyl alcohol. Using purified precipitated DNA, enrichment of the target sequences was measured by ChIP-qPCR using primers designed against the murine Gata2 intragenic enhancer.

### RNA isolation and ChIP-qPCR.

Chyle-filled mesenteric lymphatic vessels originating from mesenteric lymph nodes were identified and dissected from P5 mice. Total RNA from cultured murine primary LECs or isolated mesenteric lymphatic vessels, and formalin-fixed, methacam-fixed, paraffin-embedded (MFPE) tissue was isolated using the CellAmp Whole Transcriptome Amplification Kit and the RecoverAll Total Nucleic Acid Isolation Kit, respectively. Total RNA was reverse transcribed using iScript Reverse Transcription Supermix (Bio-Rad) or the CellAmp Whole Transcriptome Amplification Kit (Takara) according to the manufacturers’ protocols. Expression of relevant transcripts was measured by ChIP-qPCR using SYBR Green PCR Master Mix as previously described (40). Signals were normalized to corresponding GAPDH controls and are represented as relative expression ratios of the experimental samples relative to controls.

### Whole-mount IHC.

Mesentery tissues from P5 mice were dissected, fixed in 4% paraformaldehyde overnight at 4°C, washed in 1× PBS twice at 4°C for 30 minutes, and dehydrated through a methanol gradient. The samples were then incubated with antibodies against Vegfr3 (1:200) and Hdac3 (1:50) or Prox1 (1:100) overnight at 4°C. Finally, tissue samples were washed in PBS, incubated overnight at 4°C in Alexa Fluor 488– or 546/568–conjugated secondary antibodies (1:200), with or without Hoechst (1:1,000), rinsed in PBS, and then imaged using confocal microscopy.

### Evans Blue dye lymphangiography.

Evans blue dye (50 μl of 1%, 10 mg/ml), prepared in 1× PBS (pH 7.4), was injected into the left hind footpad of anesthetized mice, with the needle pointed in the dorsal direction. Fifteen minutes after injection, the mice were euthanized and dissected to examine lymphatic vessels, the thoracic duct, and lymph nodes of interest.

### Platelet count.

Approximately 100 μl of blood was drawn from each anesthetized P5 pup via intracardiac puncture and collected in 200 μl citrate-phosphate-dextrose (CPD) buffer (16 mM citric acid [anhydrous], 102 mM trisodium citrate, 18.5 mM NaH_2_PO_4_, 142 mM D-glucose, pH 7.4) as described previously (76). Platelet counts were obtained and calculated using an automated cell counter (Beckman Coulter; Ac.T 8).

### Immunoprecipitation and Western blot analysis.

Samples were homogenized in immunoprecipitation buffer (50 mM Tris-HCl pH 8.0, 150 mM NaCl, 0.5% Nonidet P-40, 1 mM EDTA, and 1 mM DTT) containing 1 mM PMSF and a protease inhibitor mixture. The homogenized samples were sonicated using a Branson 250 Digital Sonifier with 1-second-on and 1-second-off pulses at 40% power amplitude for 15 seconds. Precleared lysates were incubated with the relevant primary antibody–conjugated magnetic beads for 16 hours at 4°C. Immune complexes were collected, washed 4 times with immunoprecipitation buffer, and applied to 4%–12% SDS-polyacrylamide gels for Western blot analysis before transferring to PVDF membranes. Primary antibodies against HDAC3 (1:1,000), Tal1 (1:1,000), Gata2 (1:1,000), and Ets1/2 (1:1,000) were used and visualized by chemiluminescence using HRP-conjugated secondary antibodies. Blots were probed with α-tubulin (1:1,000) for the loading control.

### Statistics.

Statistical significance was determined using a 2-tailed Student’s *t* test, a *χ^2^* test, or a 1-way ANOVA with Sidak’s multiple comparisons test (GraphPad Prism 7.0; GraphPad Software). A *P* value of less than 0.05 was considered significant.

### Study approval.

The University of Massachusetts Medical School is accredited by the Association for Assessment and Accreditation of Laboratory Animal Care (AAALAC) International and follows the Public Health Service Policy for the Care and Use of Laboratory Animals. Animal care was provided in accordance with the procedures outlined in the *Guide for the Care and Use of Laboratory Animals* (National Academies Press, 2011). The IACUC of the University of Massachusetts Medical School approved all animal use protocols.

## Author contributions

CMT conceived the study. HPJ and CMT designed the experiments. HPJ, ZJM, and CMT performed experiments and acquired and analyzed data. MS, NDL, and JFK contributed reagents, materials, methodology, and analysis tools. HPJ, ZJM, NDL, JFK, and CMT wrote the manuscript. All authors reviewed the results and approved the final version of the manuscript.

## Supplementary Material

Supplemental data

## Version 1 10/16/2017

Electronic publication

## Version 2 11/01/2017

Print issue publication
