# Supplementary material for: Hdac3 regulates lymphovenous and lymphatic valve formation
Source: J Clin Invest. 2017 Oct 16;127(11):4193–206. doi: 10.1172/JCI92852 (PMC5663362; doi:10.1172/JCI92852)
Supplement: Supplemental data [file jci-127-92852-s001.pdf]

Figure S1, related to Figure 1

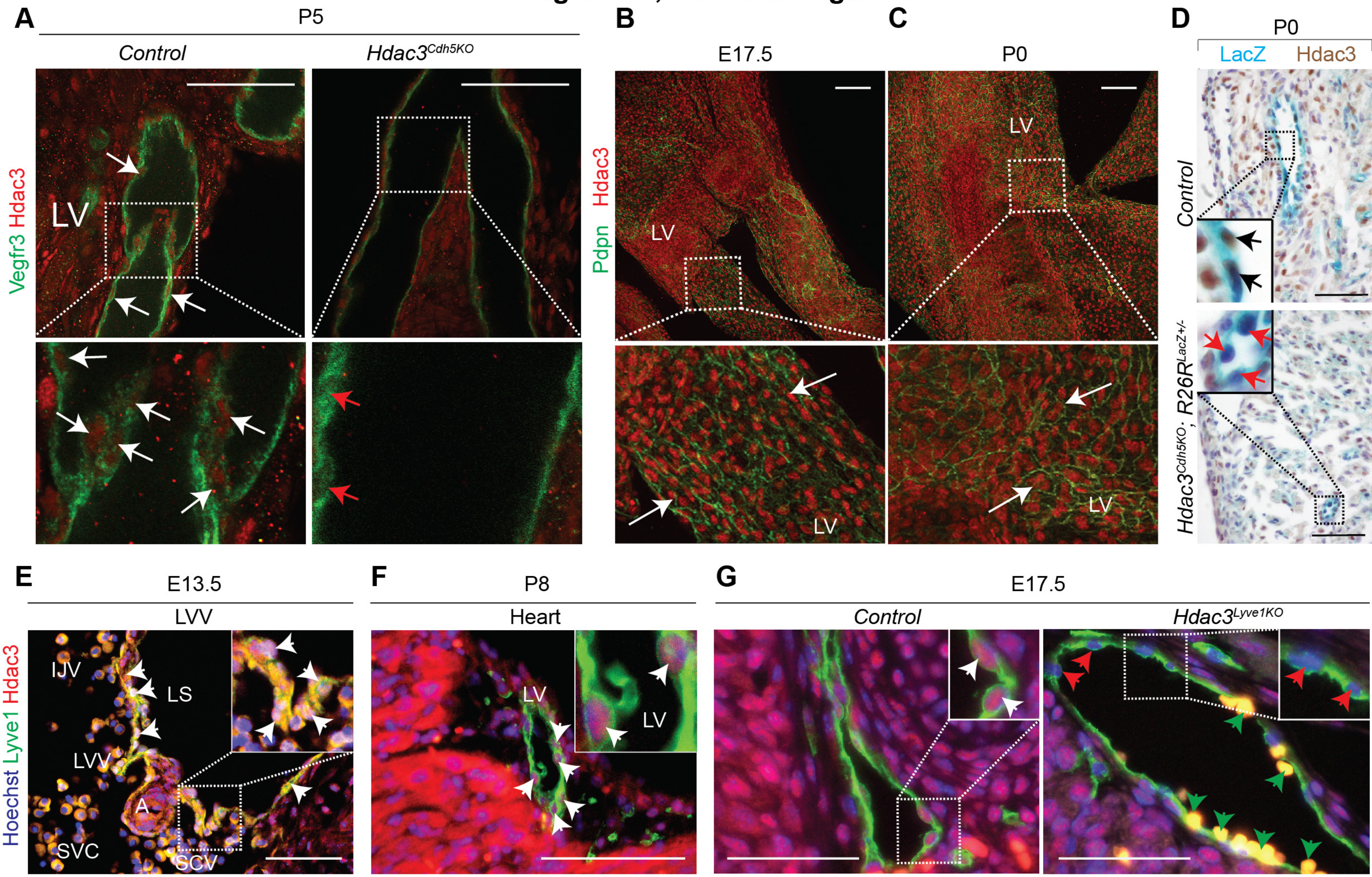

Figure S1: Hdac3 is ubiquitously expressed in the developing lymphatic vasculature - (A) Whole-mount co-immunofluorescent staining of P5 *Hdac3*^{Cdh5KO} mesenteric lymphatic vessels reveals loss of Hdac3 expression (red) in Vegfr3^{+} (green) LECs (red arrow) compared to control (white arrow). Sub-stack of Z-stack images are presented. **(B-C)** Whole-mount co-immunofluorescent staining of E17.5 (B) or P0 (C) murine mesenteric lymphatic vessels identifies Hdac3 (red) expression in Pdpn^{+} lymphatic vessels (white arrows). **(D)** Hdac3 immunostaining of LacZ stained P0 *Hdac3*^{Cdh5KO}*; R26R*$^{LacZ+/-}$ heart section show loss of Hdac3 protein expression (red arrows) in LacZ^{+} cells compared to control (black arrows). **(E-G)** Co-immunofluorescent stain for Lyve1 (lymphatic marker – green) and Hdac3 (red) shows Hdac3 expression (white arrows) in LECs forming lymphovenous valves and lymph sac at E13.5 (E), cardiac lymphatic vessel at P8 (K), and peripheral embryonic lymphatic vessels at E17.5 (G). E17.5 *Hdac3*^{Lyve1KO} peripheral lymphatic vessel (G) reveals loss of Hdac3 expression in Lyve1^{+} (green) LECs (red arrow) compared to control (white arrow). Green arrows (G) show blood-filled peripheral lymphatic vessels. LV, lymphatic valve; SVC, superior vena cava; LS, lymph sac; LVV, lymphovenous valve; A, Artery; IJV, internal jugular vein; SCV, subclavian vein. Scale bar, 100µm (A-F); Scale bar, 50µm (G).

Figure S2, related to Figure 1

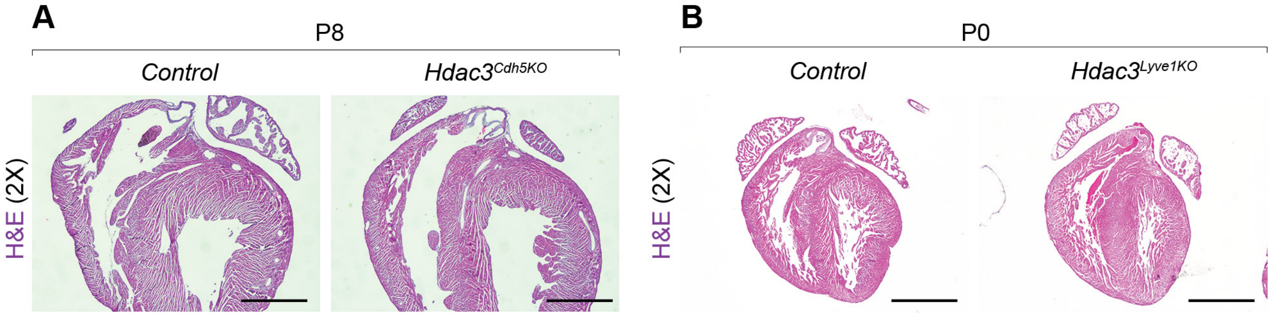

Figure S2: Endothelial Hdac3 is dispensible for myocardial development.
(A-B) H&E-stained sections show normal myocardial development in *Hdac3*^{Cdh5KO} P8 (A) or *Hdac3*^{Lyve1KO} P0 (B) hearts. Scale bar, 1000μm.

Figure S3, related to Figure 1

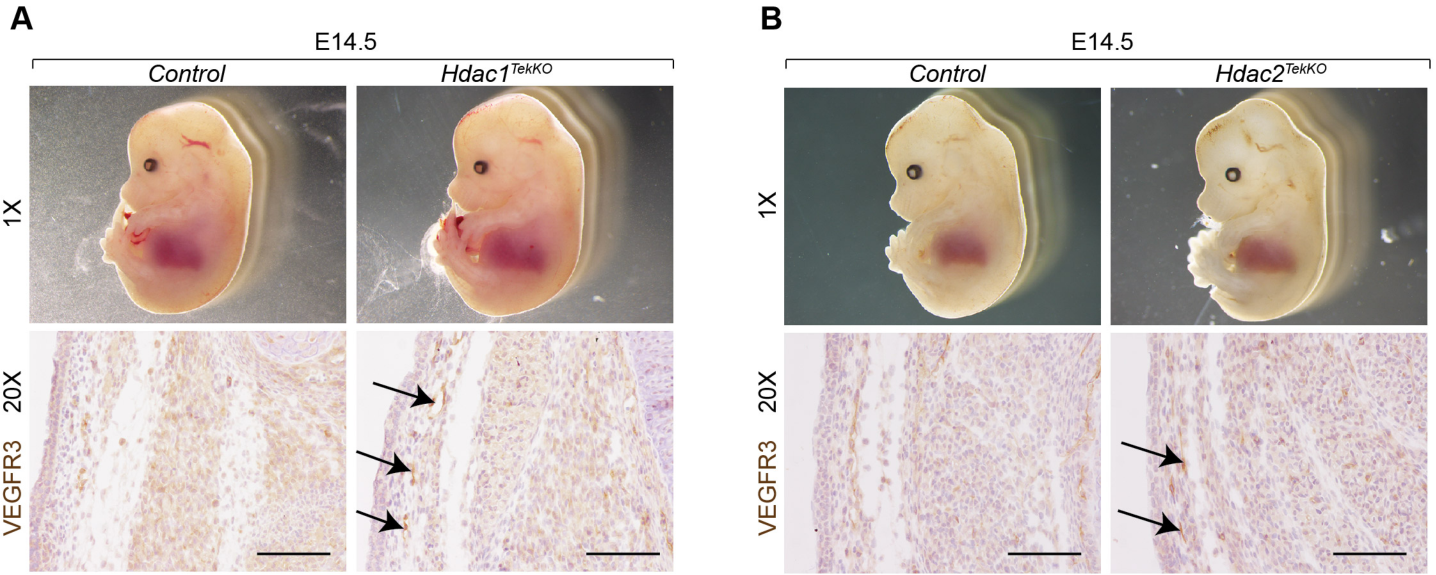

Figure S3: Dissected E14.5 *Hdac1*^{TekKO} (A) and *Hdac2*^{TekKO} (B) embryos appear normal. Lymphatic vessels in E14.5 *Hdac1*^{TekKO} and *Hdac2*^{TekKO} embryos do not show blood cells (black arrows). Scale bar, 100μm.

Figure S4, related to Figure 1

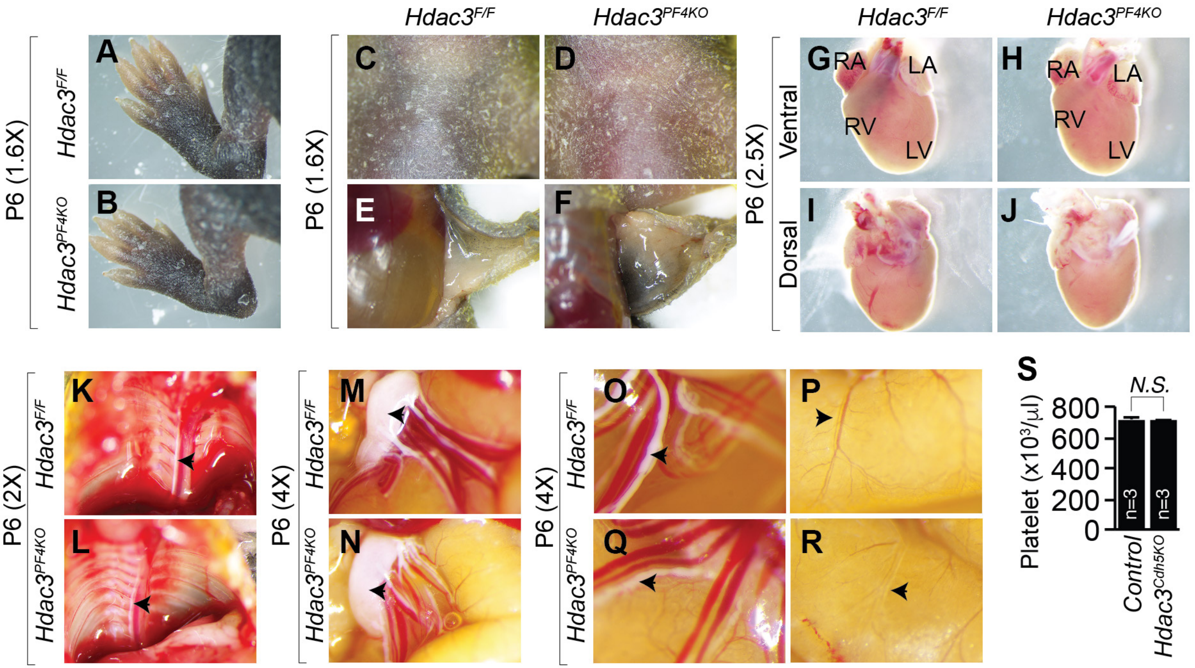

Figure S4: Platelet Hdac3 is dispensable for blood-lymphatic separation.
(A-B) *Hdac3*^{PF4KO} mice do not show hindlimb edema (B) compared to control (A). **(C-F)** Perinatal (P6) *Hdac3*^{PF4KO} mice do not display abnormal blood-filled dermal vessels (D, F) compared to control (C, E). **(G-J)** *Hdac3*^{PF4KO} heart does not show ectatic and hemorrhagic superficial vessels at P6 (H, J) compared to control (G, I). **(K-L)** Dissected P6 *Hdac3*^{PF4KO} mice do not show blood-filled thoracic duct (L, arrow) compared to chyle-filled thoracic duct (K, black arrow) in control. **(M-R)** *Hdac3*^{PF4KO} P6 mice show normal chyle-filled white mesentery lymph node (N), intestinal lymphatic vessels (Q), and mesenteric lymphatic vessels (R) compared to control (M, O, P). **(S)** *Hdac3*^{Cdh5KO} P6 mice show normal platelet counts compared to control. Data represent the mean ± SEM and are representative of three independent experiments. *P* values are determined by Student's *t* test. N.S., not significant.

Figure S5, related to Figure 2

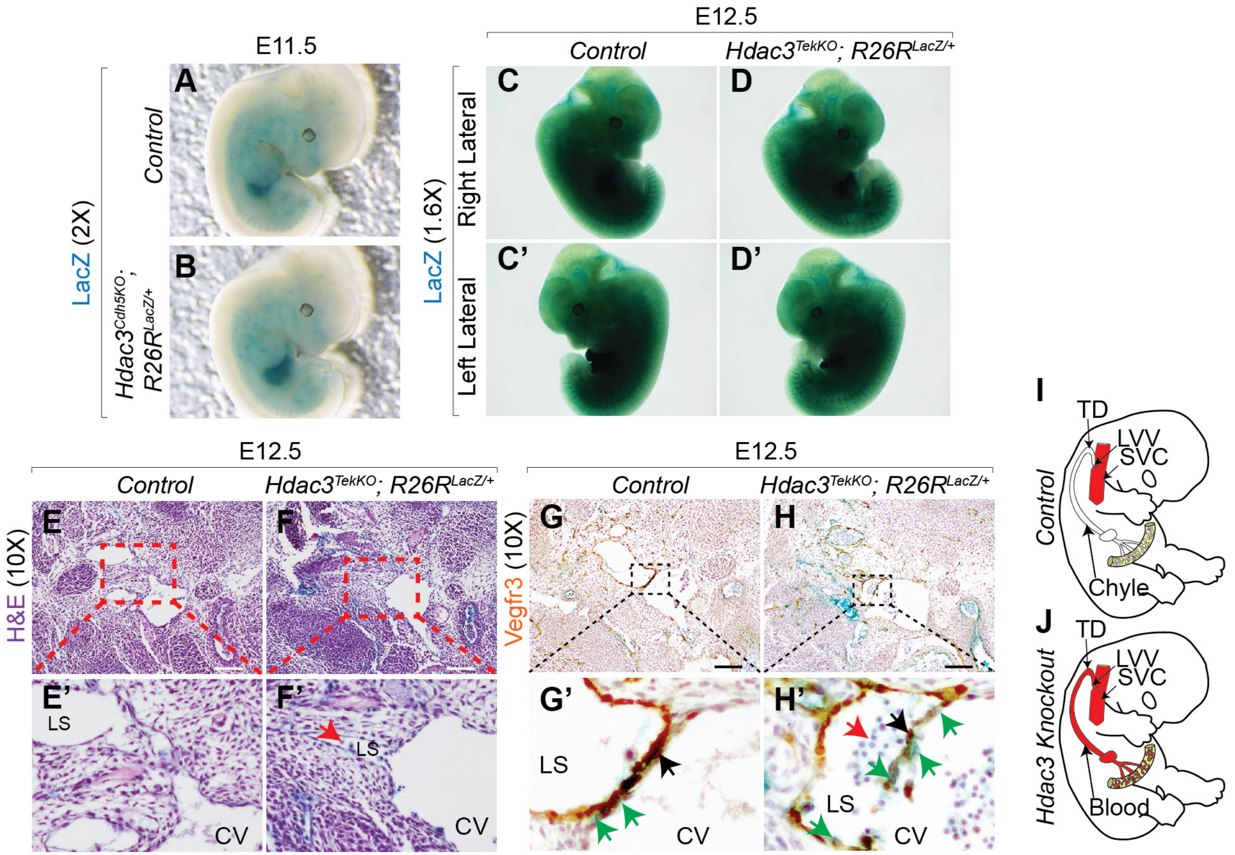

Figure S5: Endothelial Hdac3 is a critical regulator of blood-lymphatic seperation at a early stage of development.

(A-D) Whole-mount LacZ staining of control (A, C, C') and *Hdac3^{Cdh5KO}; R26R$^{LacZ/+}$* (B) or *Hdac3^{TekKO}; R26R$^{LacZ/+}$* (D, D') at E11.5 and E12.5, respectively, show similar level and pattern of LacZ expression. **(E-H)** H&E-stained transverse sections of LacZ-stained E12.5 *Hdac3^{TekKO}* (E, F) embryos reveal blood-filled lymph sacs (F', H' red arrows) compared to control (E', G'). Vegfr3 immunostaining (G-H) shows LECs-specific expression (G', H', black arrows) in LacZ^{+} (G', H', green arrows) E12.5 murine lymph sac. **(I-J)** Schematic model depicting loss of blood-lymphatic separation between SVC and TD due to defective lymphovenous valve development in Hdac3-null mice. H&E, Hematoxylin and Eosin; CV, cardinal vein; LS, lymph sac. Scale bar, 100μm.

Figure S6, related to Figure 3

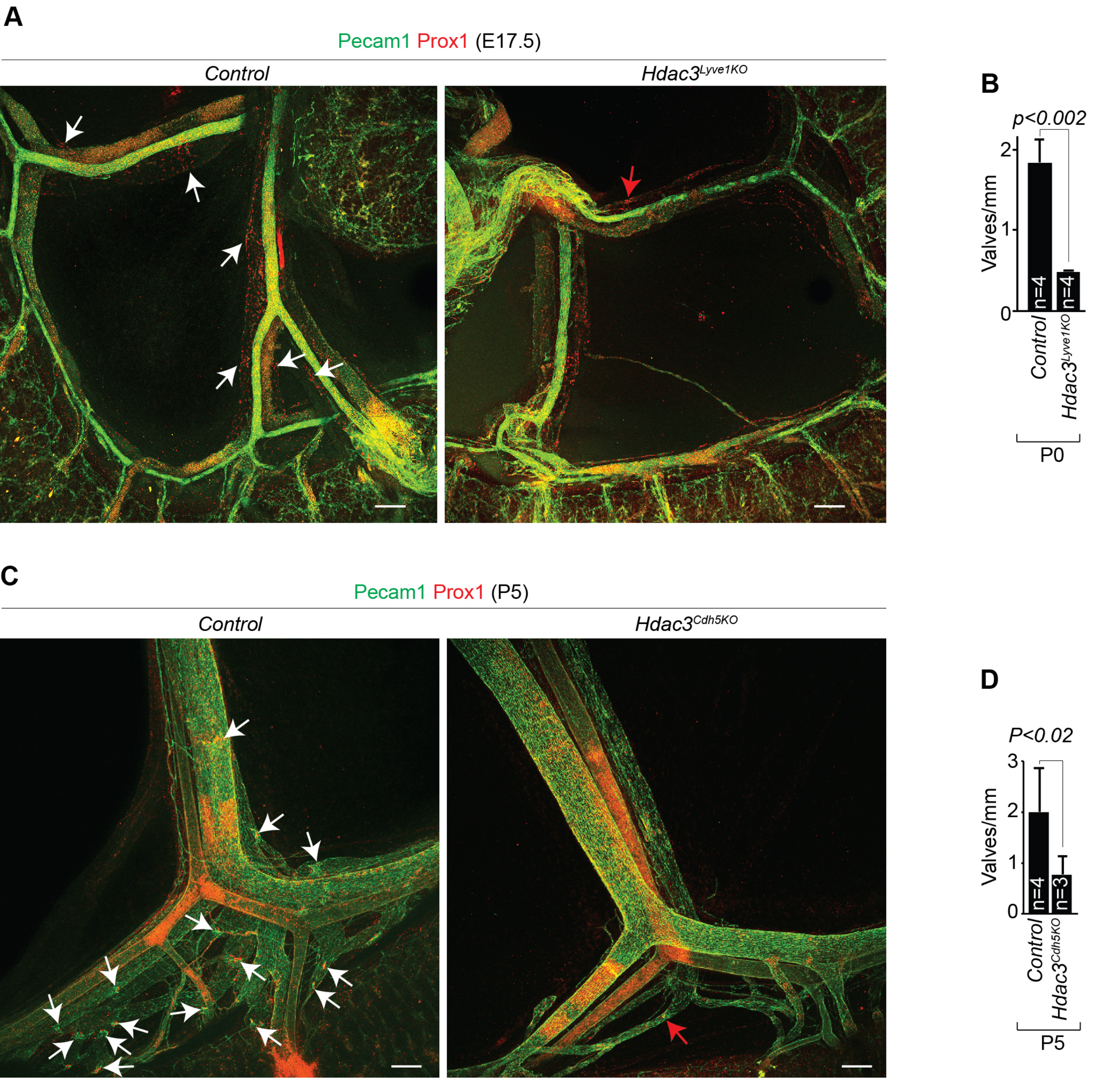

Figure S6: (A) Whole-mount co-immunofluorescent staining of E17.5 *Hdac3*^{Lyve1KO} mesenteric lymphatic vessels show reduced number of Prox1 (red) expressing valve forming territories in Pecam1^{+} (green) lymphatic vessels (red arrows) compared to control (white arrows). **(B)** Quantitation of lymphatic valves in P0 *Hdac3*^{Lyve1KO} mesenteric lymphatic vessels. **(C)** Whole-mount co-immunofluorescent staining of P5 *Hdac3*^{Cdh5KO} mesenteric lymphatic vessels lake mature Prox1 (red) expressing valves in Pecam1^{+} (green) lymphatic vessels (red arrows) compared to control (white arrows). **(D)** Quantitation of lymphatic valves in P5 *Hdac3*^{Cdh5KO} mesenteric lymphatic vessels. Data represent the mean ± SEM and are representative of three independent experiments. *P* values are determined by Student's *t* test. Scale bar, 100μm.

Figure S7, related to Figure 4

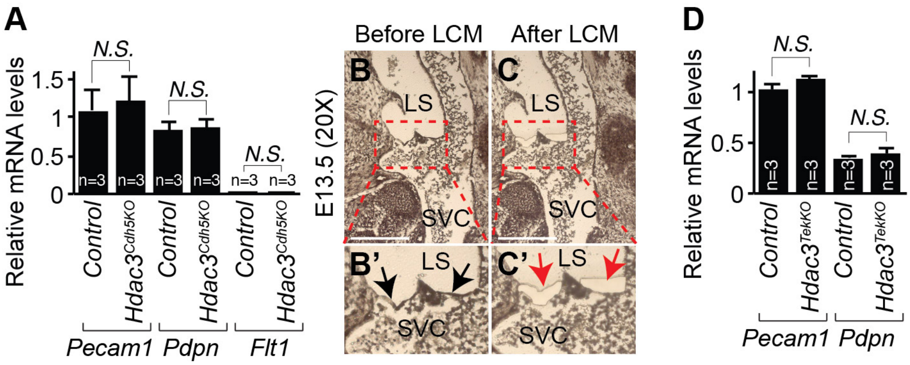

Figure S7: (A) Transcripts for *Pecam1*, *Pdpn*, and *Flt1* were detected by real-time qPCR in control and *Hdac3*^{Cdh5KO} mesenteric lymphatic vessels dissected from P5 mice. **(B-C)** Lymphovenous valves (B, B', black arrows) were microdissected (C, C', red arrows) from coronal sections of E13.5 murine embryos using laser capture. **(D)** Transcripts for *Pecam1* and *Pdpn* were detected by real-time qPCR in control and *Hdac3*^{TekKO} lymphovenous valves laser captured from E13.5 embryos. Data represent the mean ± SEM and are representative of three independent experiments. *P* values are determined by Student's *t* test. SVC, superior vena cava; LS, lymph sac; LCM, laser capture microdissection. Scale bar, 100μm.

Figure S8, related to Figure 5

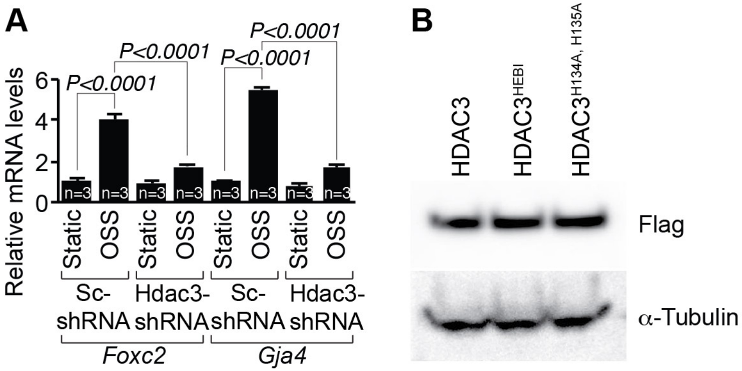

Figure S8: (A) Transcripts for *Foxc2* and *Gja4* were detected by real-time qPCR in Sc- or Hdac3-shRNA-infected LECs subjected to static or OSS and co-infected with GFP lentiviruses. **(B)** Total lysates from LECs expressing flag-tagged HDAC3, $HDAC3^{HEBI}$, or $HDAC3^{H134A,H135A}$ plasmids were analyzed by Western blot using anti-Flag antibody. α-Tubulin is shown as a loading control. Data represent the mean ± SEM and are representative of three independent experiments. *P* values are determined by one-way ANOVA using Sidak's multiple comparisons test. OSS, oscillatory shear stress; Sc, scramble.

Figure S9, related to Figures 6, 7

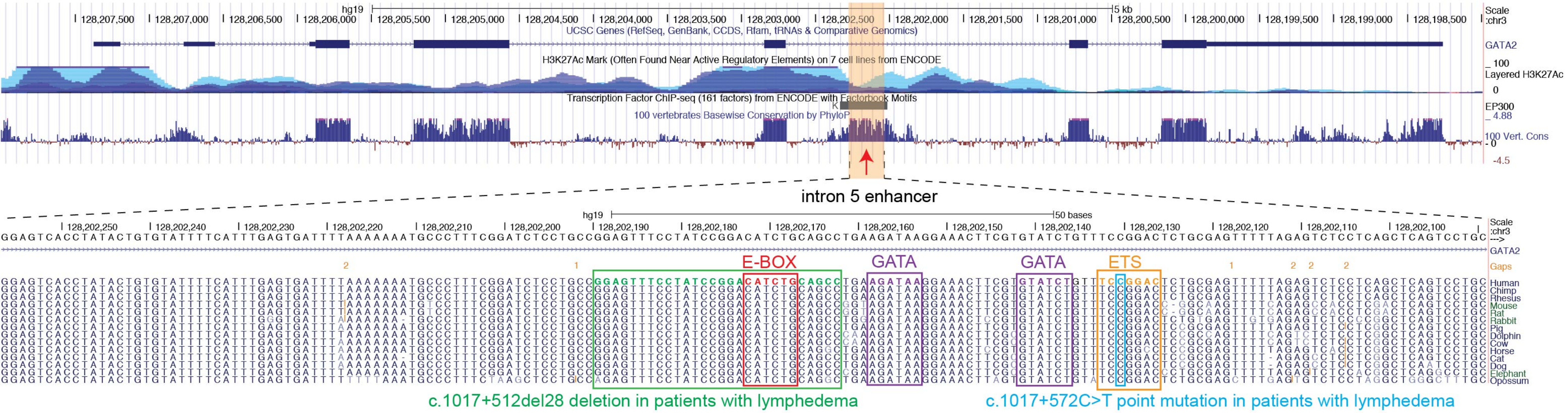

Supplemental Figure S9: UCSC genome browser view of human GATA2 locus. Based on ChIP-seq data curated by the ENCODE project consortium, GATA2 intragenic enhancer region (orange highlight) is enriched for H3K27ac and EP300. 168 bp GATA2 intragenic enhancer region shows high degree of evolutionary conservation in multiple alignments of 100 vertebrate species (red arrow). Specifically, the sequence encompassing c.1017+512del28 mutation region (green box) and c.1017+572C>T point mutation (blue box), observed in patients with lymphedema, is highly conserved among mammals. The E-box sequence (red box) is located within the c.1017+512del28 deletion region (green box) while the c.1017+572C>T point mutation is within the ETS binding site (orange box). Consensus GATA binding site (purple box) is located between E-box and ETS binding sites.

Figure S10, related to Figures 6, 7

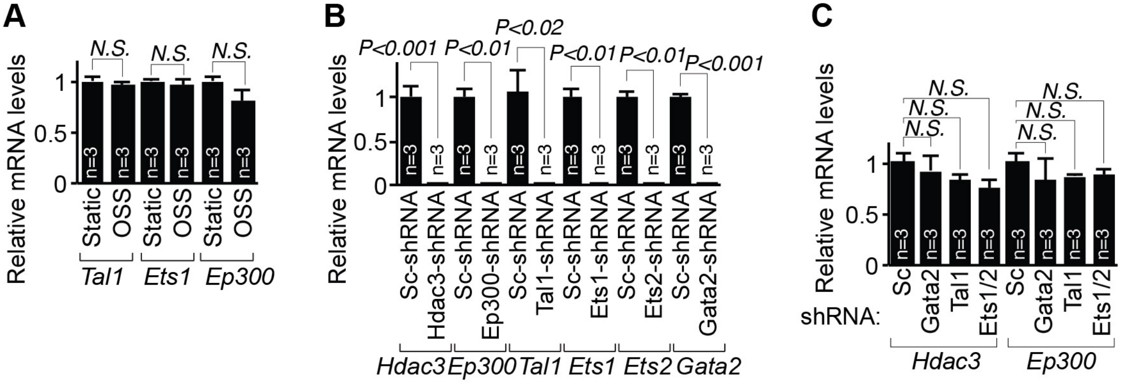

Figure S10: (A) Transcripts for *Tal1*, *Ets1*, and *Ep300* were detected by real-time qPCR in LECs subjected to static or OSS. **(B)** Transcripts for *Hdac3, Ep300, Tal1, Ets1, Ets2,* and *Gata2* were detected by real-time qPCR in Sc- or Hdac3/Ep300/Tal1/Ets1/Ets2/Gata2-shRNA-infected LECs respectively. **(C)** Transcripts for *Hdac3* and *Ep300* were detected by real-time qPCR in Sc-, Gata2-, Tal1-, or Ets1/2-shRNA-infected LECs. Data represent the mean ± SEM and are representative of three independent experiments. *P* values are determined by Student's *t* test (A, B) or by one-way ANOVA using Sidak's multiple comparisons test (C). OSS, oscillatory shear stress; Sc, scramble; N.S., not significant.

Table S1, related to figure 1

Hdac3$^{F/+}$; Tek-Cre mice were crossed with *Hdac3$^{F/+}$*

Genotype	P0
	Observed (Expected)
Hdac3$^{+/+}$	17 (16)
Hdac3$^{F/+}$	37 (32)
Hdac3$^{F/F}$	19 (16)
Hdac3$^{+/+}$;Tek-Cre	20 (16)
Hdac3$^{F/+}$;Tek-Cre	35 (32)
Hdac3$^{F/F}$;Tek-Cre	0*(16)
Total	128 (128)

* $p < 0.003$

Table S2, related to figure 1

Hdac3^F/+^; Tek-Cre mice were crossed with *Hdac3^F/F^; LacZ^-/-^*

Genotype	E12.5	E13.5	E14.5
	Observed (Expected)		
*Hdac3^F/+^; LacZ^+/-^*	7 (7.25)	11 (9.5)	4 (3.5)
*Hdac3^F/F^; LacZ^+/-^*	6 (7.25)	6 (9.5)	2 (3.5)
*Hdac3^F/+^;Tek-Cre; LacZ^+/-^*	8 (7.25)	11 (9.5)	5 (3.5)
*Hdac3^F/F^;Tek-Cre; LacZ^+/-^*	8 (7.25)	10# (9.5)	4* (3.5)
Total	29 (29)	38 (38)	15 (15)

*embryos were resorbing.

#5 out of 10 embryos were resorbing.

Table S3, related to figure 1

Hdac3$^{F/+}$*; Cdh5-Cre* mice were crossed with *Hdac3*$^{F/+}$

Genotype	P0	P8	P14
	Observed (Expected)		
Hdac3$^{+/+}$	9 (8)	17 (12)	9 (7)
Hdac3$^{F/+}$	21 (16)	18 (24)	18 (15)
Hdac3$^{F/F}$	7 (8)	14 (12)	4 (7)
Hdac3$^{+/+}$*;Cdh5-Cre*	9 (8)	14 (12)	14 (7)
Hdac3$^{F/+}$*;Cdh5-Cre*	12 (16)	30 (24)	16 (15)
Hdac3$^{F/F}$*;Cdh5-Cre*	6 (8)	3 (12)**	0 (7)*
Total	64 (64)	96 (96)	61 (61)

$^{**}P < 0.03$ $^{*}P < 0.05$

Table S4, related to figure 1

Hdac3$^{F/+}$; Lyve1-Cre mice crossed with Hdac3$^{F/F}$LacZ$^{-/-}$

Genotype	P0	P5
	Observed	(Expected)
Hdac3$^{F/+}$; LacZ$^{+/-}$	3 (7.5)	7 (7)
Hdac3$^{F/F}$; LacZ$^{+/-}$	6 (7.5)	12 (7)
Hdac3$^{F/+}$; Lyve1-Cre; LacZ$^{+/-}$	13 (7.5)	9 (7)
Hdac3$^{F/F}$; Lyve1-Cre; LacZ$^{+/-}$	8 (7.5)[a]	0 (7)**
Total	30	28

a = 2 Dead ** $P < 0.02$

Supplemental Table S5: Phenotypes of mice lacking endothelial Hdac3

Genotype	Age	X^2 test	P-value
Blood-filled dermal lymphatic vessels			
Hdac3^{TekKO}	E13.5	*Hdac3*^{TekKO} (6/6): *Control* (0/4)	*P = 0.0016*
Hdac3^{Cdh5KO}	P6	*Hdac3*^{Cdh5KO} (3/3): *Control* (0/3)	*P = 0.0143*
Blood-filled cardiac lymphatic vessels			
Hdac3^{Cdh5KO}	P0	*Hdac3*^{Cdh5KO} (2/3): *Control* (0/4)	*P = 0.0533*
Hdac3^{Cdh5KO}	P8	*Hdac3*^{Cdh5KO} (3/3): *Control* (0/3)	*P = 0.0143*
Hdac3^{Lyve1KO}	P0	*Hdac3*^{Lyve1KO} (3/3): *Control* (0/3)	*P = 0.0143*
Blood-filled mesenteric lymphatic vessels			
Hdac3^{Cdh5KO}	P5	*Hdac3* ^{Cdh5KO} (1/6): *Control* (0/3)	*P = 0.4533*
Hdac3^{Cdh5KO}	P6	*Hdac3*^{Cdh5KO} (3/4): *Control* (0/4)	*P = 0.0285*
Blood-filled thoracic duct			
Hdac3^{Cdh5KO}	P5	*Hdac3*^{Cdh5KO} (3/3): *Control* (0/3)	*P = 0.0143*
Defective mesenteric lymphatic valve development			
Hdac3^{Cdh5KO}	P5	*Hdac3* ^{Cdh5KO} (3/3): *Control* (0/4)	*P = 0.0082*
Hdac3^{Levy1KO}	E17.5	*Hdac3*^{Cdh5KO} (3/3): *Control* (0/3)	*P = 0.0143*
Hdac3^{Lyve1KO}	P0	*Hdac3*^{Lyve1KO} (4/4): *Control* (0/4)	*P = 0.0047*
Defective lymphovenous valve development			
Hdac3^{TekKO}	E13.5	*Hdac3*^{TekKO} (3/3): *Control* (0/3)	*P = 0.0143*
Hdac3^{Cdh5KO}	E17.5	*Hdac3*^{Cdh5KO} (3/3): *Control* (0/3)	*P = 0.0143*

Supplemental Table S6:

Antibody	Company	Catalogue Number	Species	Clonality	Final Conc.
Hdac1	Abcam	ab19845	Rabbit	Polyclonal	0.91-1μg/ml
Prox1	Abcam	ab101851	Rabbit	Polyclonal	10μg/ml
Prox1	Abcam	ab37128	Rabbit	Polyclonal	1:100
Foxc2	Abcam	ab5060	Goat	Polyclonal	5μg/ml
H3K27ac	Abcam	Ab4729	Rabbit	Polyclonal	10μg/ml
alpha-SMA	Abcam	ab5694	Rabbit	Polyclonal	2μg/ml
Tal1	Santa Cruz Biotechnologies	sc-12984	Goat	Polyclonal	200ng/ml
Ets1/2	Santa Cruz Biotechnologies	sc-374509	Mouse	Monoclonal	200ng/ml
Ep300	Santa Cruz Biotechnologies	sc-585	Rabbit	Polyclonal	200ng/ml
Gata2	Santa Cruz Biotechnologies	sc-9008	Rabbit	Polyclonal	2μg/ml
Emcn	Santa Cruz Biotechnologies	sc-53941	Rat	Monoclonal	4μg/ml
Hdac3	Santa Cruz Biotechnologies	sc-11417	Rabbit	Polyclonal	2μg/ml
Vegfr3	R&D Systems	AF743	Goat	Polyclonal	1μg/ml
Lyve1	R&D Systems	AF2125	Goat	Polyclonal	2μg/ml
Nrp1	R&D Systems	AF566	Goat	Polyclonal	4μg/ml
Pdpn	R&D Systems	AF3244	Goat	Polyclonal	4μg/ml
Alexa Fluor 488	Life Technologies	A-21208	Donkey	Polyclonal	4μg/ml
Alexa Fluor 488	Life Technologies	A-11055	Donkey	Polyclonal	4μg/ml
Alexa Fluor 568	Life Technologies	A10042	Donkey	Polyclonal	4μg/ml
Donkey anti-Goat TRITC	Santa Cruz Biotechnologies	Sc-3855	Donkey	N/A	0.8μg/ml
Hdac2	Life Technologies	51-5100	Rabbit	Polyclonal	2.5μg/ml
Flag	Sigma	F-3165	Mouse	Monoclonal	200ng/ml
CD31	BD Pharmingen	550274	Rat	Monoclonal	78.125ng/ul
Pdpn	Hybridoma Bank	8.1.1-c	Hamster	Polyclonal	1.4μg/ml
Itga9	R&D Systems	AF3827	Goat	Polyclonal	0.2μg/ml
Lyve1	Abcam	Ab14917	Rabbit	Polyclonal	10μg/ml
Anti-syrian hamster Immunopure FITC	Life Technologies	31587	Rabbit	Polyclonal	4μg/ml
Anti- Goat Alexa Fluor 568	Life Technologies	A-11079	Rabbit	Polyclonal	4μg/ml
